# Exosomal miR-93-3p targets EIF4EBP1 to regulate macrophage polarization and accelerate wound healing post-anal fistula surgery

**DOI:** 10.3389/fphar.2025.1599633

**Published:** 2025-08-18

**Authors:** Wei Zhang, Wenzhe Feng, Junhong Chen, Ruoxi Cao, Xi Chen

**Affiliations:** ^1^ First Clinical Medical College, Shaanxi University of Chinese Medicine, Xianyang, China; ^2^ Department of Anorectal Surgery, Affiliated Hospital of Shaanxi University of Chinese Medicine, Xianyang, China

**Keywords:** anal fistula, wound healing, miR-93-3p, target gene EIF4EBP1, MP

## Abstract

**Background:**

Delayed wound healing following anal fistula (AF) surgery remains a clinical challenge. This study endeavors to identify and validate key exosomal miRNAs that regulate postoperative inflammation after AF surgery by integrating multi-omics analyses with functional assays, and to elucidate the molecular mechanisms by which these miRNAs and their target genes influence macrophage M1/M2 polarization.

**Methods:**

15 patients undergoing AF surgery were randomized to three groups. The negative control group received sterile Vaseline gauze dressings, the positive control cohort took Kangfuxin Solution, and the treatment cohort received Wugu Qilin Ointment. Wound exudates were collected postoperatively and exosomes were isolated via ultracentrifugation. Total RNA was extracted through the TRIzol method, followed by miRNA microarray analysis to identify differentially expressed miRNAs (DEMs). Candidate miRNAs were validated via qPCR to identify those significantly linked to the therapeutic efficacy of the traditional Chinese medicine (TCM) method of Euriching Pus for Tissue Growth. *In vitro*, the differentiation of THP-1 cells into macrophages was employed via PMA. The MP were verified by flow cytometry (FC), qPCR and Western blotting (WB). Potential miRNA target genes were predicted using TargetScan before Gene Ontology (GO) functional annotation and Kyoto Encyclopedia of Genes and Genomes (KEGG) pathway enrichment analysis. The direct interplay between miRNA and its target gene was verified through a dual-luciferase reporter (DLR) assay.

**Results:**

Microarray analysis and qPCR validation identified miR-93-3p as the most significantly DEMs. miR-93-3p overexpression markedly downregulated M1 macrophage marker CD86 and pro-inflammation cytokines IL-1β, IL-6, and TNF-α, while upregulating M2 markers Arg-1, CD206, and anti-inflammation cytokines IL-10 and TGF-β in functional assays. Conversely, miR-93-3p suppression exhibited the opposite effect. WB analysis confirmed that miR-93-3p bidirectionally regulated CD86, Arg-1, and CD206 protein expression. Bioinformatic analysis suggested that miR-93-3p possibly targets EIF4EBP1, thereby modulating biological processes like inflammatory response, cellular metabolism, and MP. This regulatory relationship was unveiled through DLR assays, proving that miR-93-3p specifically suppresses EIF4EBP1 expression.

**Conclusion:**

This study is the first to elucidate the molecular mechanism by which the TCM therapeutic approach of Euriching Pus for Tissue Growth promotes M2 MP through the exosomal miR-93-3p/EIF4EBP1 axis, and theoretically supports the formulation of new exosome-based miRNA treatment strategies for postoperative anti-inflammatory treatment in AF.

## 1 Introduction

Anal fistula (AF) features the formation of an aberrant tract between the rectum or anal canal and the perianal skin, often giving rise to chronic inflammation, recurrent infections, and a potential risk of malignant transformation (Geldof et al., 2022). Epidemiological data suggest that the incidence of AF is nearly 3.6% (Huang et al., 2022). To date, surgical intervention remains the most effective treatment modality (Shi et al., 2021). However, the postoperative wound is typically large and left open, with considerable exudate, often leading to delayed healing (Singh et al., 2021; Farag et al., 2019), which severely affects patients’ physical and psychological wellbeing and urgently warrants a solution.

The administration of antibiotics postoperatively can effectively reduce recurrence rates (Mocanu et al., 2019). According to traditional Chinese medicine (TCM), delayed wound healing after AF surgery primarily arises from the persistence of damp-heat pathogenic factors (with disease pathogenesis involving damp-heat, qi stagnation, and blood stasis). The damp-heat syndrome manifests as localized heat (indicative of inflammation), tissue edema (reflecting impaired water metabolism), and excessive secretions (like thick exudate from the postoperative wound) (Yu et al., 2025). The TCM external therapeutic approach of Euriching Pus for Tissue Growth has demonstrated efficacy in suppressing inflammation in postoperative wounds of AF (Liu et al., 2024) and has shown remarkable therapeutic effects for chronic large-area wounds, like those resulting from AF surgery, diabetic foot ulcers, and burns (Lu et al., 2022; Fan et al., 2022), particularly during the inflammatory phase. A distinguishing feature of this therapy is its reinterpretation of “pus” as containing beneficial components that promote healing (e.g., macrophages and fibroblasts) (Hassel et al., 2018), fundamentally differing from the purulent fluid defined in Western medicine. Based on this theory, the Wugu Qilin Ointment has been developed and proven effective in clinical applications (Zheng and Wang, 2013; Cao and Feng, 2023).

The fistulous tract is hallmarked by persistent inflammation and aberrant epithelialization, with its microenvironment characterized by the accumulation of pro-inflammatory cytokines like TNF-α and IL-1β (Wang et al., 2025). Macrophages, as key immune regulators (Kim and Nair, 2019), initially adopt the M1 phenotype to release pro-inflammatory mediators and clear necrotic tissue, and subsequently polarize toward the M2 phenotype to resolve inflammation and facilitate tissue repair (Yunna et al., 2020).

Exosomes, nano-sized vesicles (40–160 nm) secreted by cells, modulate the activity of target cells by delivering functional molecules including RNAs, proteins, and lipids (Dai et al., 2020). As a principal subpopulation of extracellular vesicles (EVs), exosomes, together with microvesicles and apoptotic bodies, constitute the broader EV system (Zhao et al., 2021; Mathieu et al., 2019). They are released by varied cells like macrophages and endothelial cells, into bodily fluids including blood and cerebrospinal fluid (van Niel et al., 2018; Liao et al., 2014; Saunderson et al., 2014). Exosome function is cell-specific: For example, tolerated macrophage exosomes can induce the differentiation of regulatory T cells (Tregs), thereby inhibiting excessive inflammation (Théry et al., 2002), while tumor epithelial cell exosomal integrins (such as ITGβ4) can drive organ-specific metastasis (Wu et al., 2023). Exosomes upregulate anti-inflammation factors through the let-7b/TLR4 pathway and promote macrophage polarization (MP) of M2, thereby suppressing inflammation and accelerating wound healing (Ti et al., 2015). As intercellular communication vectors, exosomes regulate recipient cell function by transporting miRNAs (Corrado et al., 2013; Yu et al., 2014; Huang-Doran et al., 2017). For example, exosomal miR-124 enhances the anti-inflammatory effects of astrocytes and facilitates neural repair (Lee et al., 2014). miRNAs mediate gene expression via binding to the 3′-untranslated regions (3′-UTR) of target genes. For example, miR-222-3p facilitates M2 polarization by targeting Bim, thereby alleviating inflammation and accelerating wound healing in diabetic patients (Xia et al., 2023).

However, none has elucidated the influence of exosomal miRNAs and their target genes in mediating MP to alleviate inflammation in postoperative AF wounds. Therefore, this study aims to isolate exosomes from postoperative wound exudates, termed “euriched pus” in the context of Euriching Pus for Tissue Growth, to identify differentially expressed exosomal miRNAs related to this therapy, and to explore the molecular mechanisms through which exosomal miRNAs and their target genes regulate wound healing after AF surgery. This investigation seeks to elucidate the anti-inflammatory mechanism by which the TCM external treatment Euriching Pus for Tissue Growth promotes wound healing and to lay a scientific foundation for the treatment of postoperative AF wounds.

## 2 Materials and methods

**FIGURE 1:**
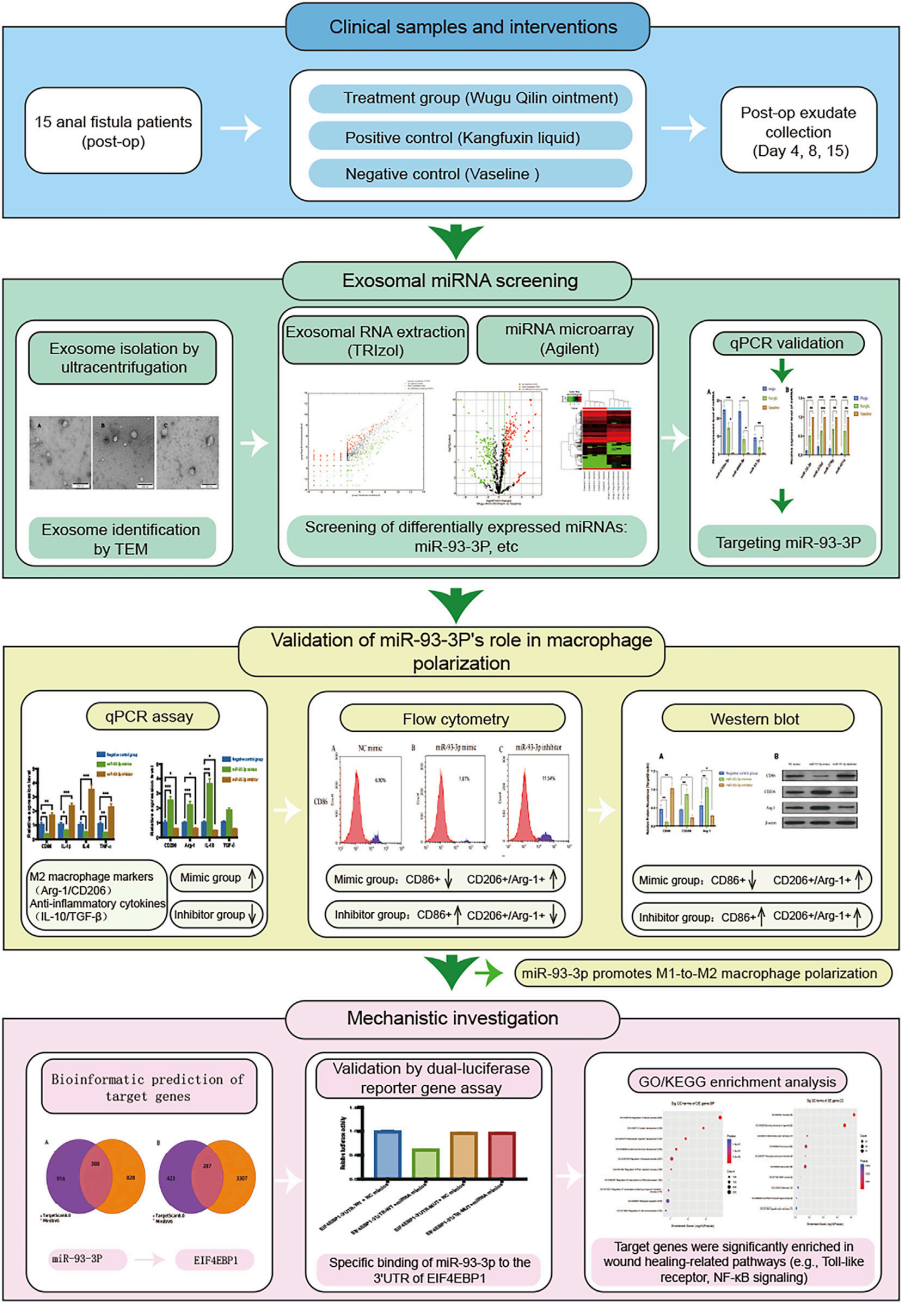
Technology Roadmap.

Experimental Design. (The overall experimental workflow, including clinical sample processing, *in vitro* functional validation, and mechanistic exploration, is summarized in Figure 1).

### 2.1 Patient selection and grouping

This study strictly followed the inclusion and exclusion criteria (Supplementary Material S1) and enrolled a total of 15 patients who underwent low anal fistula resection. The patients were randomized into three groups (n = 5 per group): the experimental group (treated with Wugu Qilin Ointment-soaked gauze), the positive control group (treated with Kangfuxin Solution-soaked gauze), and the negative control group (treated with Vaseline-soaked gauze). All interventions commenced immediately after surgery, using a standardized dressing change protocol (twice daily with sterile dressing coverage and fixation), and continued until complete wound healing.

### 2.2 Medicine preparation

Wugu Qilin Ointment was prepared by the Preparation Center of the Affiliated Hospital of Shaanxi University of Chinese Medicine following the topical preparation standards outlined in the *Pharmacopoeia of the People’s Republic of China* (2020 edition). The ointment is composed of *Chrysomya megacephala* (30 g), Daemonorops draco (30 g), Coptis chinensis (30 g), and Arnebia euchroma (15 g), with a drug ratio of *C. megacephala*: Daemonorops draco: Coptis chinensis: Arnebia euchroma = 2:2:2:1 (w/w). All medicinal materials were verified using taxonomic databases (Supplementary Material S2). The preparation process was: *C. megacephala*, Coptis chinensis, and Arnebia euchroma were soaked in 400 mL of sesame oil for 3 days, then simmered over low heat until the herbs were charred and desiccated, which was followed by filtration. Daemonorops draco was then added and dissolved, and beeswax was gently heated and incorporated. After cooling, 40 pieces of 3 × 10 cm sterilized gauze were immersed in the ointment for 4 hours and stored at 4 °C for later use. Orthogonal fingerprint profiling was performed via liquid chromatography-mass spectrometry (LC-MS) in both positive and negative ion modes. The top ten characteristic peaks were selected based on scoring and used for total ion chromatogram (TIC) analysis (Supplementary Figures S1, S2). In positive ion mode, the detection of 7-hydroxy-3-phenyl-chromen-4-one (m/z 239.0703, retention time 381 s), a flavonoid component accounting for 42.7% of the peak area, suggests it as a major active constituent, consistent with the known bioactive compounds in Daemonorops draco and Coptis chinensis. The detection of Carnitine (m/z 162.1125) indicates the presence of amino acid metabolites. In negative ion mode, alpha-linolenic acid (m/z 277.2173) and linoleic acid (m/z 279.2330) together accounted for 68.3% of the total lipid content, confirming that the sesame oil matrix components are well preserved (Detailed data provided in Supplementary Material S3, 4; The ConPhyMP checklist can be found in Supplementary Material S4, 5).

Kangfuxin Solution Gauze: Sterile gauze strips (3 cm × 10 cm) were immersed in Kangfuxin Solution (manufactured by Sichuan Good Doctor Panxi Pharmaceutical Co., Ltd.; Approval No.: National Medicine Permit No. Z51021834).

Vaseline Gauze: Commercial Vaseline gauze (3 cm × 10 cm × 10 strips per pack) produced by Henan Yadu Industrial Co., Ltd. was used (Registration No.: National Food and Medical Device (Permit) 2014 No. 3641199).

### 2.3 Ethics and informed consent

The study protocol was reviewed and approved by the Ethics Committee of the Affiliated Hospital of Shaanxi University of Chinese Medicine (Approval No.: SZFYIEC-YJ-2023-[126]). Every participant gave informed consent in writing.

### 2.4 Experimental methods

#### 2.4.1 Exosome isolation from wound exudate and miRNA expression profiling

Wound dressings were collected on postoperative days 4, 8, and 15. After PBS extraction, centrifugation (12,000 rpm for 15 min), and 0.22 μm filtration, samples were stored at −80 °C. Exosomes were isolated using ultracentrifugation (500×g for 5 min → 2000×g for 10 min → 10,000×g for 30 min → 100,000×g for 70 min), and identified by transmission electron microscopy with phosphotungstic acid negative staining. Exosomal RNA was extracted using the TRIzol LS method (The quantification of sample RNA is provided in Attachment 6). The Agilent miRNA microarray (8 × 60k) was used to detect the expression profiles of 2,549 miRNAs. The screening criteria were |log_2_FC| ≥ 1 and FDR <0.05. Detailed experimental procedures, major reagents, and instruments were presented in Supplementary Material S7.

#### 2.4.2 qPCR validation of DEMs

Following RNA extraction via the TRIzol approach and reverse transcription into cDNA, miRNA expression was assessed through qPCR (U6-normalized, triplicate wells). The 2^-ΔΔCT calculation method was applied for relative quantification using a real-time fluorescent PCR platform, and melting curves were analyzed. The specific experimental procedure was provided in Supplementary Material S8.

#### 2.4.3 Effect of exosome-derived miR-93-3p on MP

THP-1 cells were thawed and maintained in RPMI-1640 medium containing 10% fetal bovine serum (FBS) at 37 °C with 5% CO_2_. At the logarithmic growth phase, they were passaged at a 1:3 ratio. Macrophage differentiation was stimulated via 100 ng/mL PMA. Cells were then split into miR-93-3p mimic (100 nM mimic), inhibitor (100 nM inhibitor), and negative control (100 nM NC mimic) groups (n = 3/group). The specific experimental procedures, main reagents, and instruments were presented in Supplementary Material S9.

##### 2.4.3.1 Quantitative PCR detection of miR-93-3p expression in macrophages

Total RNA from macrophages was isolated via TRIzol and subsequently reverse-transcribed into cDNA. qPCR analysis was performed in triplicate with U6 serving as the internal control. The conditions consisted of an initial denaturation at 95 °C for 10 min, 40 cycles of 95 °C for 10 s, and 60 °C for 1 min, with fluorescence acquisition. Melting curve analysis was carried out.

##### 2.4.3.2 Verification of MP via flow cytometry (FC), qPCR, and Western blotting (WB)

FC was employed to assess MP markers (M1: CD86; M2: Arg-1, CD206). mRNA expression of M1-related CD86, IL-1β, IL-6, TNF-α and M2-associated Arg-1, CD206, IL-10, and TGF-β was quantified via qPCR, with β-actin being the internal reference. CD86, Arg-1, and CD206 protein expression was analyzed through WB.

#### 2.4.4 Target gene prediction

Candidate miRNA target genes were forecast based on TargetScan 8.0 (analyzing seed sequences and conserved 3′UTRs and the miRDB V6 database. TargetScan conducts species-specific prediction using independent modules for human and murine datasets, whereas miRDB utilizes a machine learning algorithm optimized for target gene interaction prediction.

#### 2.4.5 Dual-luciferase reporter (DLR) assay

HEK-293T cells were cultured in DMEM containing 10% FBS and 1% penicillin-streptomycin and passaged according to standard protocols. For transfection, the Lipofectamine 2000 system was employed: 1 μg of plasmid DNA or 2.5 μL of siRNA was mixed with 3 μL of transfection reagent in Opti-MEM medium for 20 min, followed by transfection into logarithmically growing cells (1 × 10^5^ cells/well). Four experimental groups were established: Group A (WT 3′UTR + NC mimics), Group B (WT 3′UTR + miRNA mimics), Group C (Mutant 3′UTR + NC mimics), and Group D (Mutant 3′UTR + miRNA mimics). After 48 h of transfection, miRNA regulation was assessed through a DLR assay, with results shown in the ratio of Renilla luciferase to firefly luciferase activity. The specific experimental procedures, main reagents, and instruments were provided in Supplementary Material S10.

#### 2.4.6 Pathway enrichment analysis

Gene Ontology (GO) and Kyoto Encyclopedia of Genes and Genomes (KEGG) enrichment analyses were carried out on the target genes of DEMs to identify functional pathways implicated in macrophage phenotype regulation. The analyses were enabled by the clusterProfiler package (functions: enrichGO and enrichPathway) in R 4.4.3, and results were visualized via dot plots.

### 2.5 Data analysis

Statistical analyses were enabled by SPSS 26.0 and R 4.3.1. Measurement data are shown in mean ± standard deviation (SD) (x̄ ± s), and categorical ones were examined via the chi-square test. Regarding miRNA expression profiling, high-throughput sequencing data were analyzed with DESeq2 or edgeR, while microarray data were processed using the limma package. DEMs were identified based on |log_2_FC| ≥ 1 and FDR <0.05, with results presented via volcano plots and hierarchical clustering. Inter-group comparisons were conducted using ANOVA or t-tests, with P < 0.05 denoting statistical significance. Every experiment was carried out three times independently. *P < 0.05, **P < 0.01, ***P < 0.001.

## 3 Results

### 3.1 Identification of DEMs linked to the Euriching Pus for Tissue Growth method

#### 3.1.1 General characteristics of patients with low AF

15 low AF individuals were enrolled. They underwent surgical excision of the low AF. Statistically significant differences were absent across three treatment groups in baseline clinical characteristics (P > 0.05) (Table 1).

**TABLE 1:** General clinical data of patients with low AF.

Basic information	Wugu Qilin ointment group	Kangfuxin solution group	Vaseline group
Number of Patients	5	5	5
Age (y, ±SD)	34.20 ± 8.04	35.20 ± 7.73	41.40 ± 9.76
Male Sex	60%	60%	60%

#### 3.1.2 Morphological Characteristics of wound exudate-derived exosomes under TEM

**FIGURE 2:**
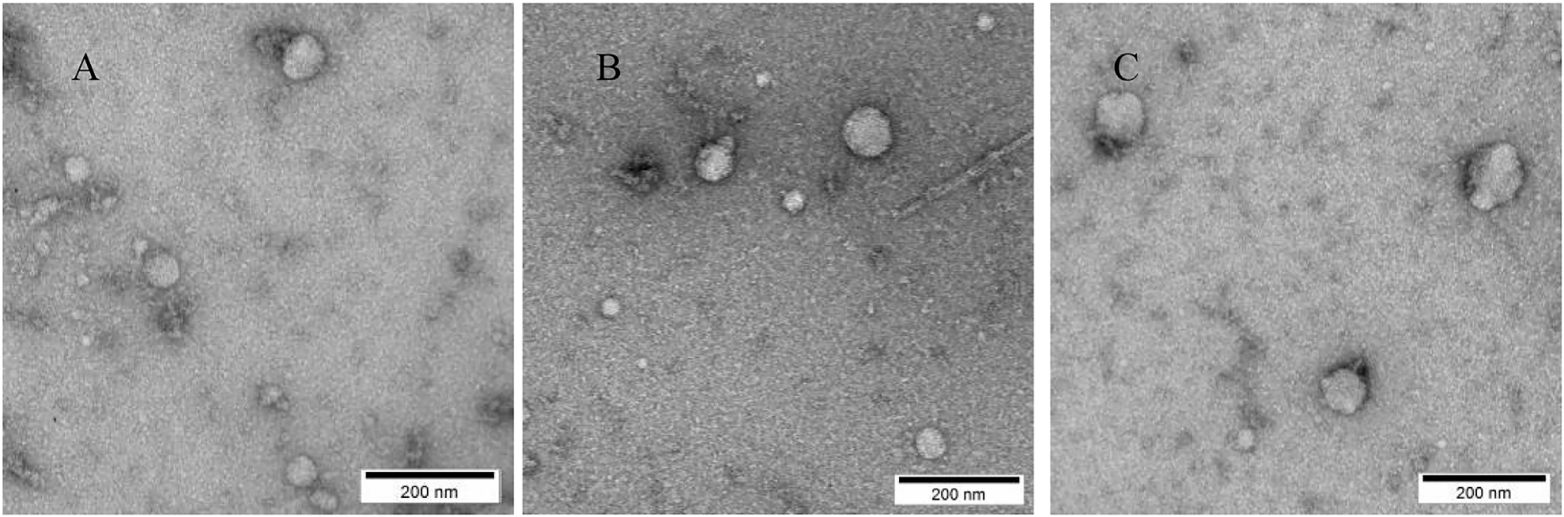
Morphological Characteristics of Exosomes Observed by TEM; **(A)** Representative cup-shaped morphology of exosomes derived from the Wugu Qilin Ointment group; **(B)** Morphological characteristics of exosomes in the Kangfuxin Solution group; **(C)** Morphology of exosomes in the Vaseline group. Scale bar: 200 nm.

As illustrated in Figure 2, vesicles extracted from all three treatment groups exhibited classical exosomal morphology, characterized by a bilayer membrane and a predominantly spherical or ovoid structure.

#### 3.1.3 The differential expression profile of exosomal miRNAs Reveals an intervention-specific regulatory pattern

**FIGURE 3:**
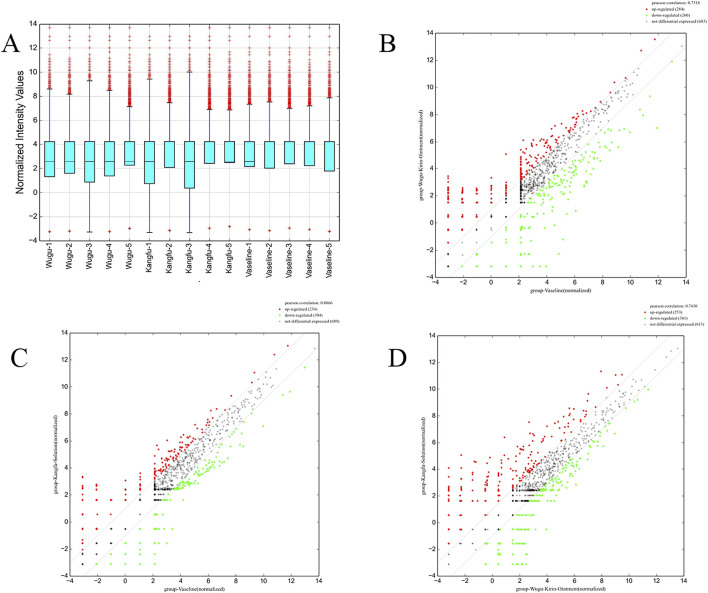
Comparative Analysis of miRNA Expression Profiles Among Treatment Groups; **(A)** Box plots of normalized signal intensities across the Wugu Qilin Ointment, Kangfuxin Solution, and Vaseline groups (n = 5). The y-axis represents log_2_-transformed normalized signal intensities; blue boxes indicate interquartile ranges (IQR), purple lines indicate 1.5× IQR, and red crosses denote outliers; **(B–D)** Scatter plots of DEMs across groups. Red and green dots denote markedly upregulated and downregulated miRNAs (|log_2_FC| ≥ 1, FDR <0.05); grey dashed lines indicate the no-change threshold (log_2_FC = 0). All data were derived from normalized sequencing results.

The miRNA expression profiles among the three groups demonstrated high concordance, as evidenced by elevated Pearson correlation coefficients (Figures 3B–D). However, differential expression analysis revealed that the median expression level of miRNAs in the Wugu Qilin Ointment cohort was notably higher in contrast to the control cohort (Figure 3A). Principal component analysis confirmed the clustering of data within each group, indicating the reliability of the experimental data. Based on the reliable data, a systematic analysis of differential expression was subsequently conducted.

**FIGURE 4:**
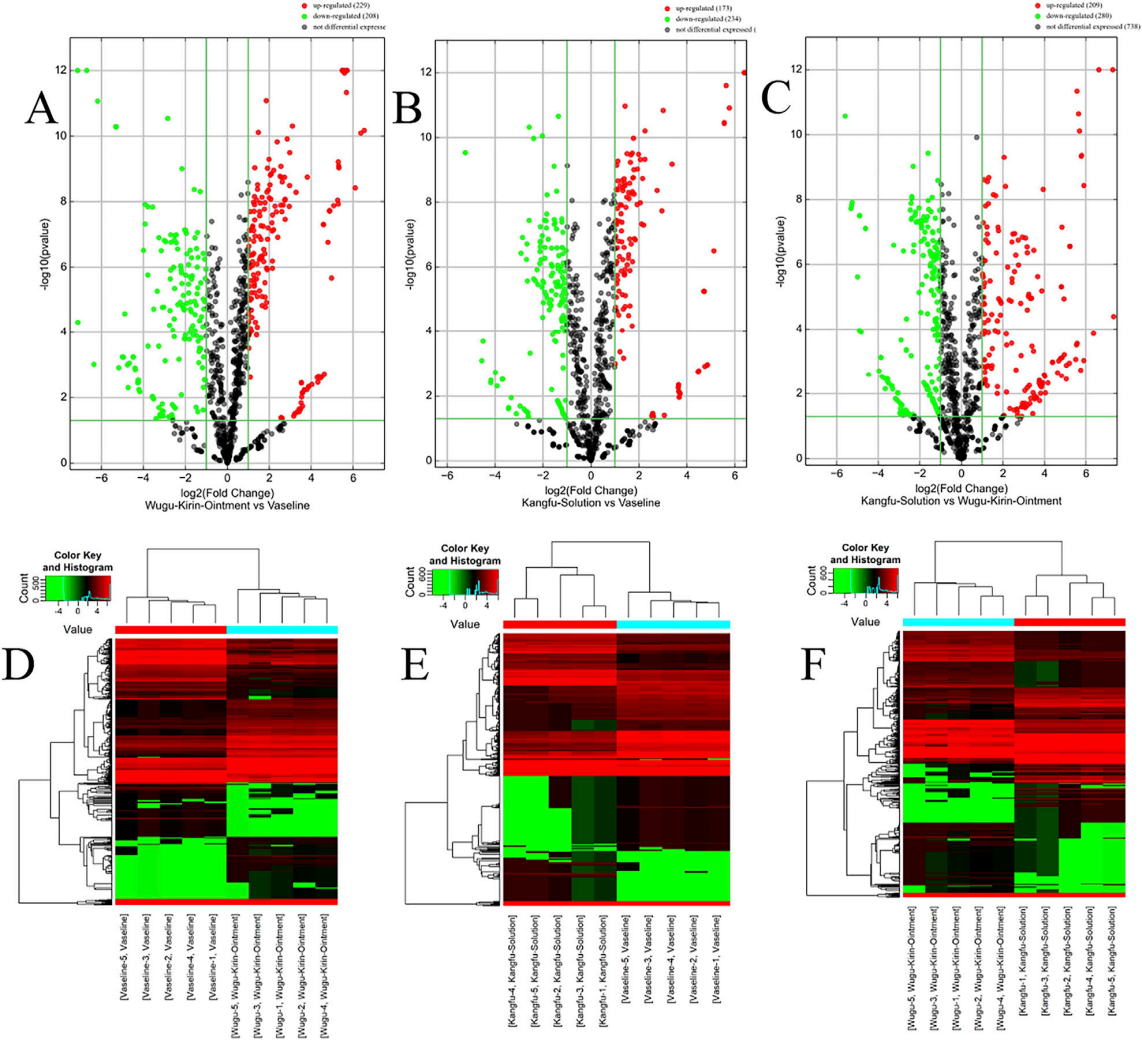
Volcano Plots and Heatmaps; **(A–C)** Volcano plots presenting differential miRNA expression between groups (x-axis: log_2_(FC); y-axis: log_10_ (p-value)); **(D–F)** Corresponding heatmaps of DEMs. Red and green indicate upregulation and downregulation, respectively.

Differential expression analysis identified 437 significantly dysregulated miRNAs between the Wugu Qilin Ointment and Vaseline groups (229 upregulated, 208 downregulated); 407 miRNAs between the Kangfuxin Solution and Vaseline groups (173 upregulated, 234 downregulated); and 489 miRNAs between the Kangfuxin Solution and Wugu Qilin Ointment groups (209 upregulated, 280 downregulated) (Figures 4A–C). In the volcano plots, red and green dots denote significantly upregulated and downregulated miRNAs, respectively. Hierarchical clustering (Figures 4D–F) revealed distinct expression patterns among the three groups. The heatmaps further highlighted intervention-specific regulatory features. Full data are detailed in Supplementary Material S11, 12.

#### 3.1.4 qPCR validation of DEMs

**FIGURE 5:**
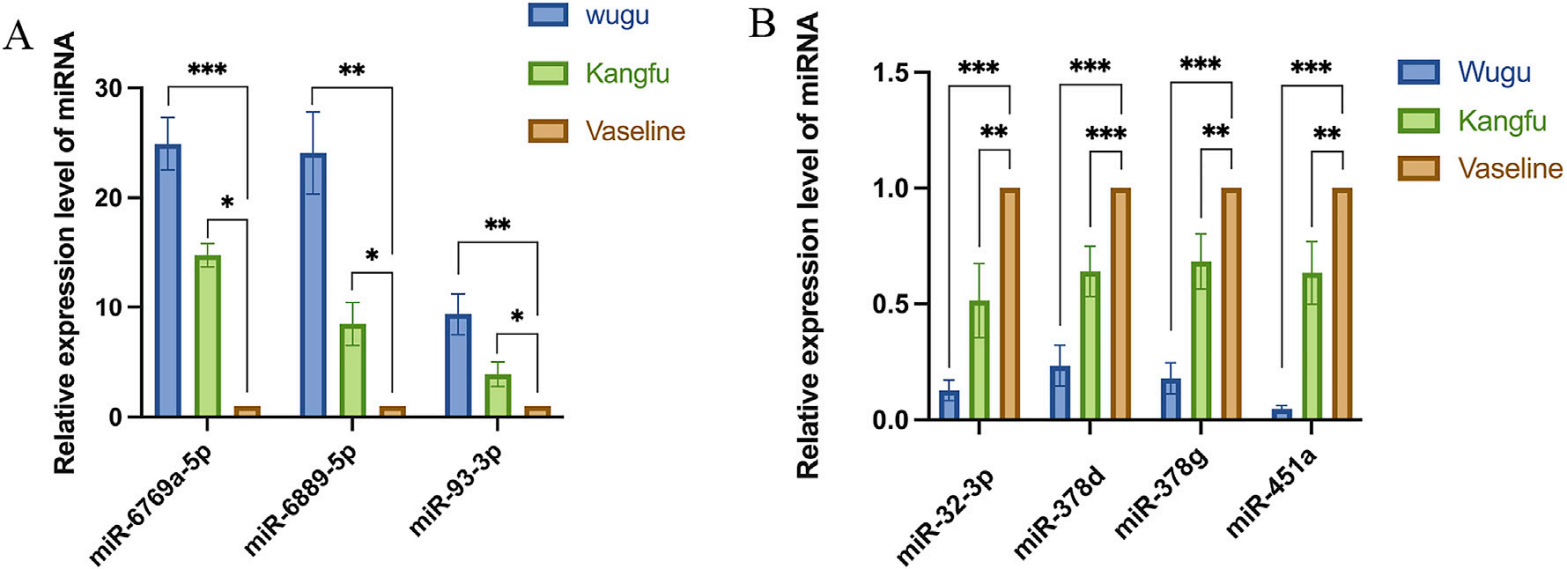
qPCR Validation of Differential miRNA Expression. **(A)** Significantly upregulated miRNAs: relative expression levels of miR-6769a-5p, miR-6889-5p, and miR-93-3p; **(B)** Significantly downregulated miRNAs: relative expression levels of miR-32-3p, miR-378d, miR-378g and miR-451a.

Through intersection analysis of differentially expressed miRNAs (DEMs) among the three groups, we identified 13 significantly altered miRNAs, which were subsequently validated by qPCR. The validation results demonstrated that seven miRNAs exhibited expression patterns completely consistent with the high-throughput sequencing data: miR-6769a-5p, miR-6889-5p, and miR-93-3p (Figure 5A) showed significant upregulation, while miR-32-3p, miR-378d, miR-378g, and miR-451a (Figure 5B) were significantly downregulated. Based on both the magnitude of differential expression (miR-93-3p displayed the highest Fold Change value) and its well-documented biological functions, we selected miR-93-3p as the core target for subsequent functional investigations to elucidate its molecular regulatory mechanisms in wound healing.

### 3.2 miR-93-3p promotes macrophage polarization: evidence from qPCR, flow cytometry, and Western blot assays

**FIGURE 6:**
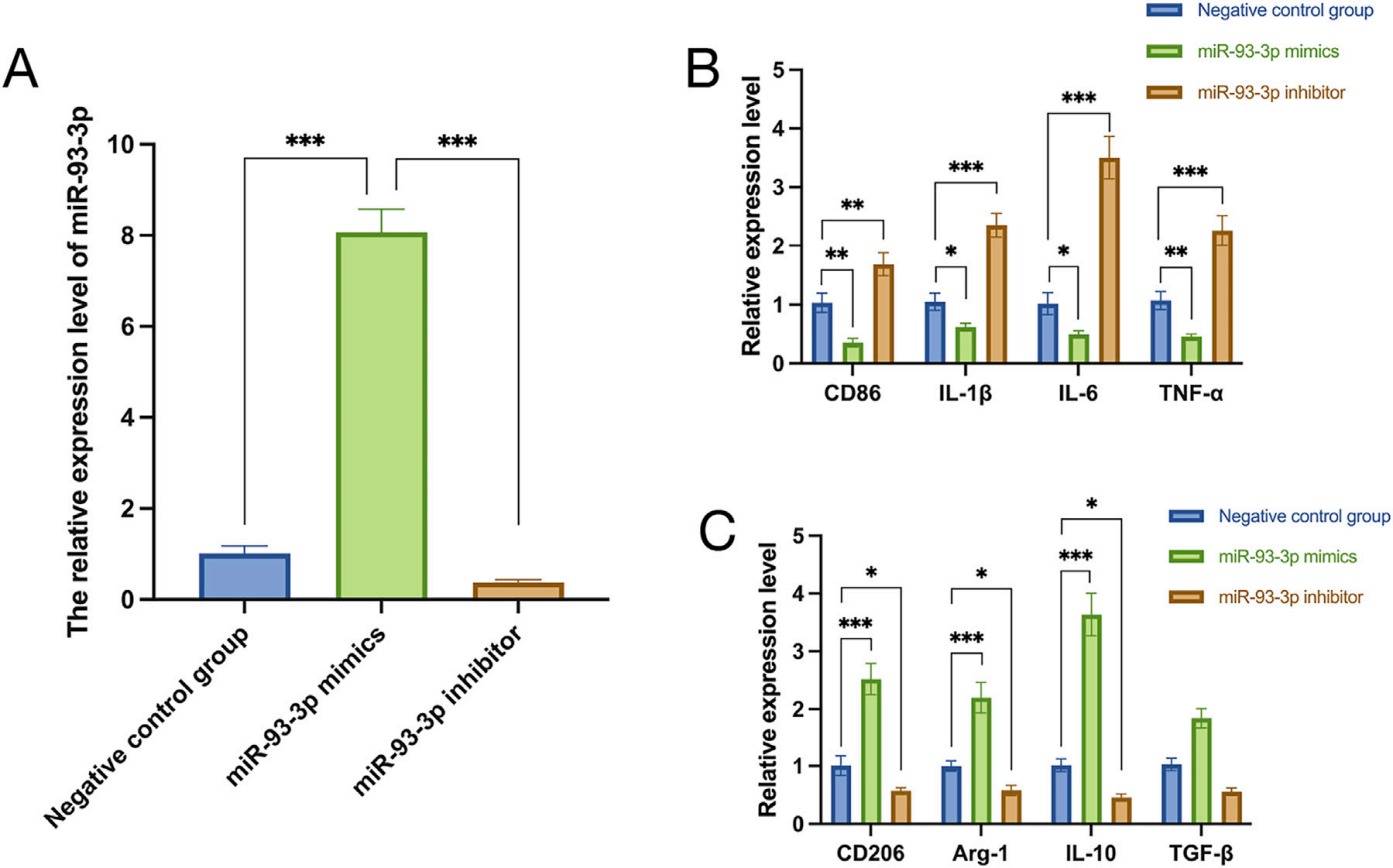
**(A)** miR-93-3p expression. **(B)** Expression of M1 marker CD86 and pro-inflammation cytokines (IL-1β, IL-6, TNF-α). **(C)** Expression of M2 markers CD206, Arg-1 and anti-inflammation cytokines (IL-10, TGF-β).

To unravel how miR-93-3p influences macrophage phenotypes, qPCR was employed to assess its relative expression in macrophages from every cohort. In contrast to the negative control cohort, the miR-93-3p mimic cohort exhibited elevated expression, whereas the inhibitor group displayed reduced levels (Figure 6A). These findings indicate that the transfection of miR-93-3p mimics and inhibitors successfully achieved effective modulation of miR-93-3p expression levels in macrophages. Specifically, the mimic group exhibited a marked upregulation of miR-93-3p expression, whereas the inhibitor group demonstrated a significant downregulation. These results confirm the stability and reliability of the experimental intervention model, thereby laying a sound foundation for subsequent investigations into the functional role of miR-93-3p in regulating macrophage polarization.

**FIGURE 7:**
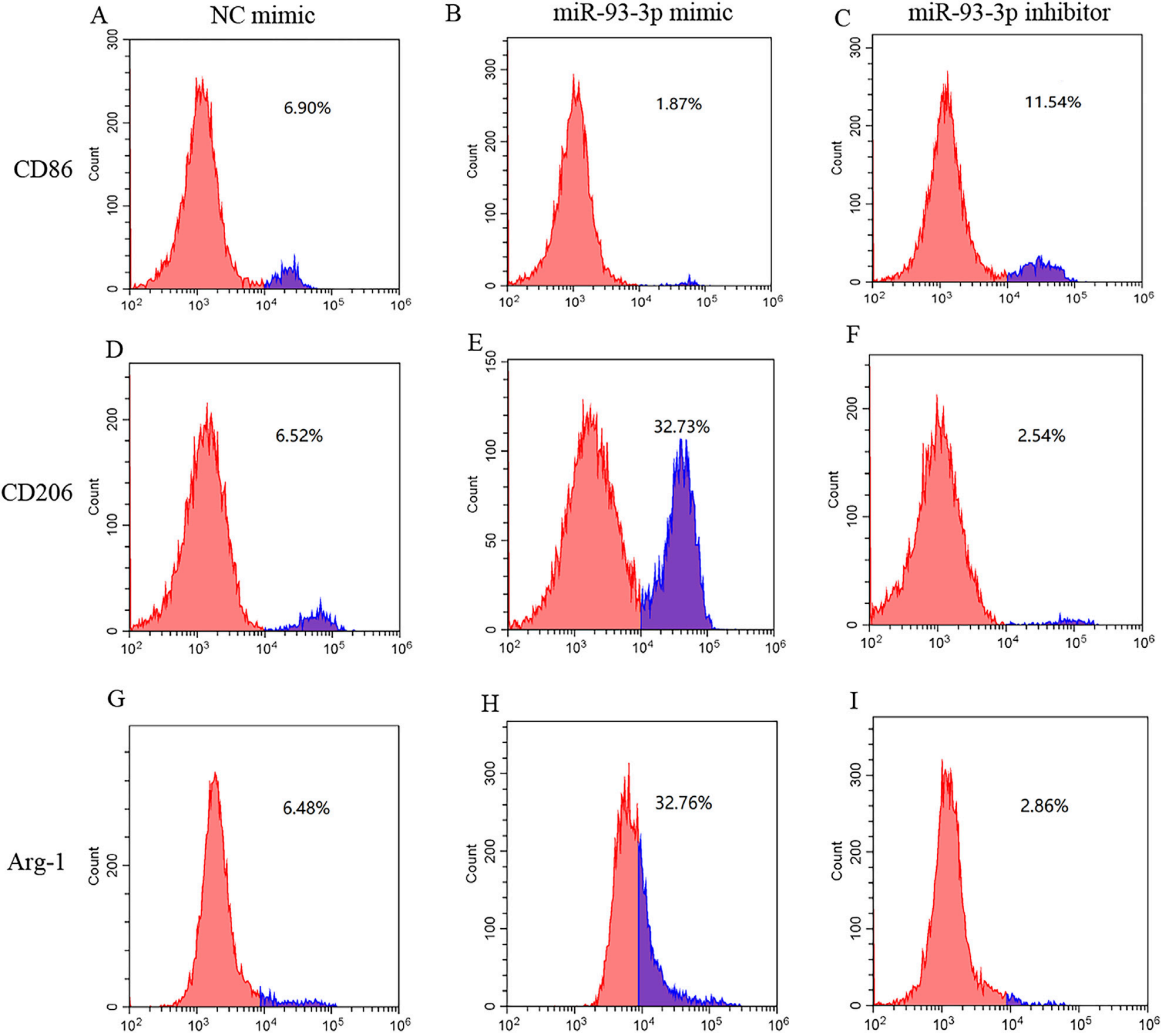
Regulatory Effect of miR-93-3p on MP; **(A–C)** CD86 (M1 marker) expression: **(A)** NC group; **(B)** mimic group; **(C)** inhibitor group. **(D–F)** CD206 (M2 marker) expression: **(D)** NC group; **(E)** mimic group; **(F)** inhibitor group. **(G–I)** Arg-1 (M2 marker) expression: **(G)** NC group; **(H)** mimic group; **(I)** inhibitor group.

**FIGURE 8:**
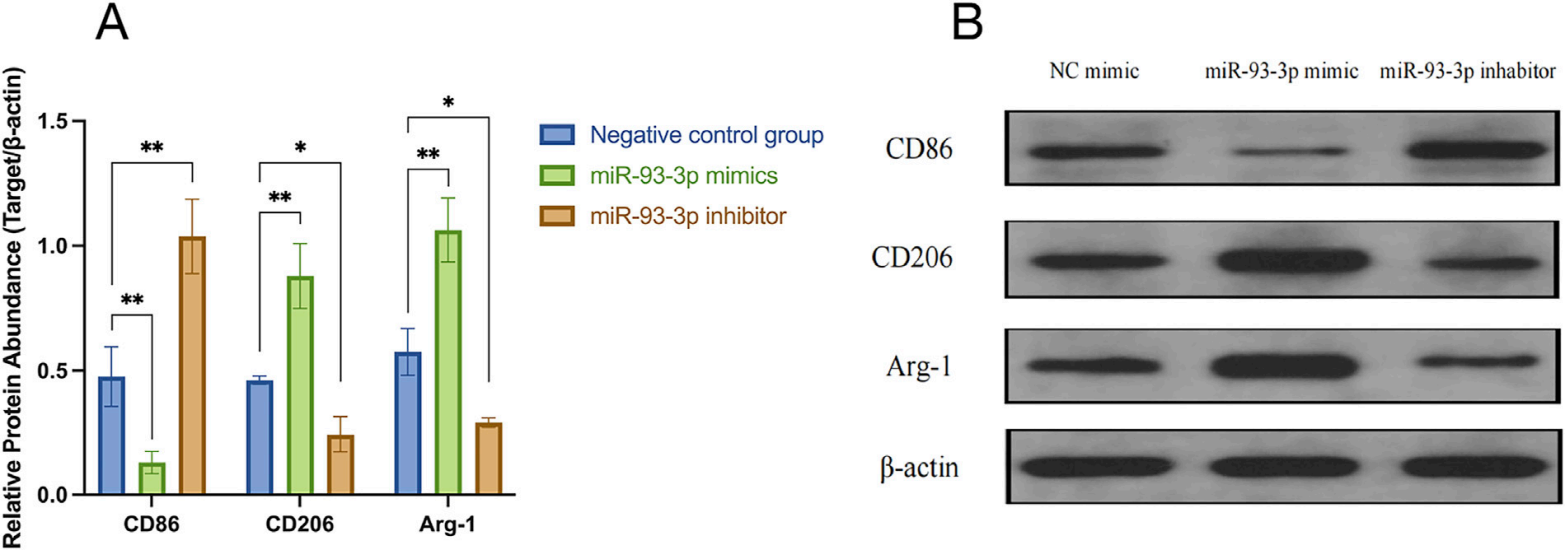
Regulatory Effects of miR-93-3p on MP-related Protein Expression. **(A)** Bar graph illustrating the relative expression levels (Target/β-actin ratio) of CD86 (M1 marker), CD206, and Arg-1 (M2 markers). **(B)** WB bands confirming the differential expression of CD86, CD206, Arg-1, and internal control β-actin across the three groups.

qPCR analysis revealed that the miR-93-3p mimic group exhibited reduced expression of M1 markers (CD86) and pro-inflammation cytokines (IL-1β, IL-6, TNF-α), alongside upregulation of M2 markers (Arg-1, CD206) and anti-inflammation cytokines (IL-10, TGF-β). However, the inhibitor group demonstrated the opposite pattern (Figures 6B,C). To further elucidate how miR-93-3p regulates MP, the expression of M1 marker CD86 and M2 markers Arg-1 and CD206 was assessed via FC (Figure 7). In contrast to the NC cohort, the miR-93-3p mimic group exhibited markedly fewer CD86^+^ cells (1.87% vs. 6.90%), while CD206^+^ and Arg-1^+^ cell proportions were significantly elevated (32.73%/32.76% vs. 6.52%/6.48%). The inhibitor group displayed the opposite trend. Meanwhile, the expression levels of key polarization-associated proteins were examined using WB (Figure 8). Quantitative analysis (Figure 8A) showed that relative to the NC group, the miR-93-3p mimic group exhibited marked downregulation of CD86 and upregulation of the Arg-1 and CD206, whereas the inhibitor cohort displayed the opposite trend. WB bands (Figure 8B) visually confirmed the downregulation of CD86 and upregulation of Arg-1 and CD206 in the mimic group, with the inverse pattern observed in the inhibitor group.

These findings confirm at the protein level that miR-93-3p promotes the MP from an M1 to an M2 phenotype through a bidirectional regulatory mechanism. Therefore, it was concluded that miR-93-3p facilitates the resolution of wound-associated inflammation. In the subsequent investigation, our study aimed to further elucidate its underlying regulatory mechanisms by examining its specific target gene interactions.

### 3.3 Target gene prediction of DEMs

**FIGURE 9:**
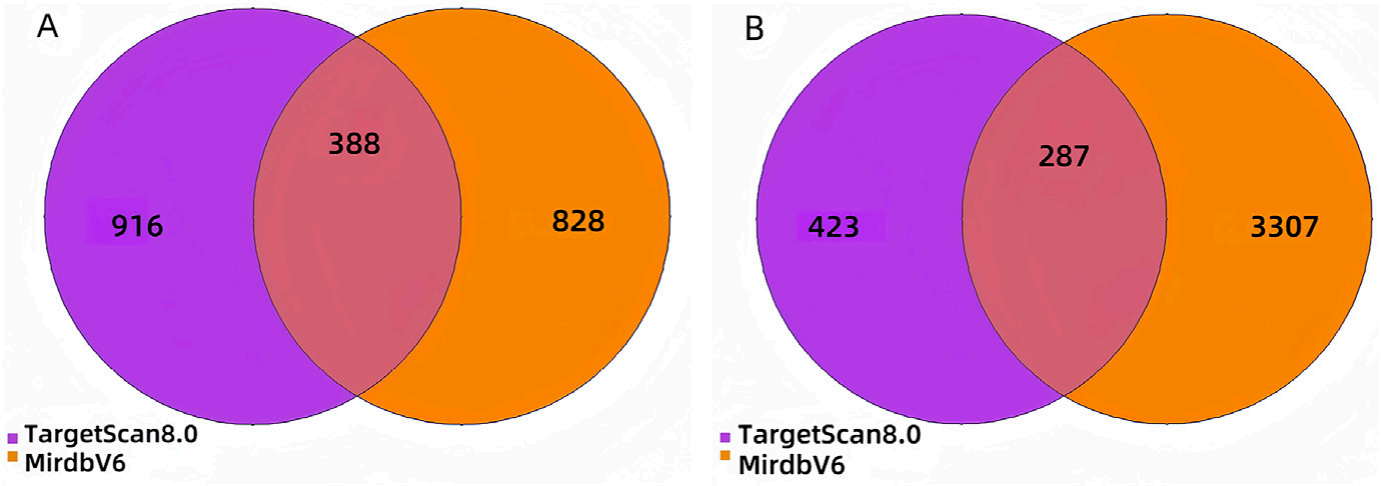
Venn Diagram. **(A)**: Target genes of upregulated DEMs; **(B)** Target genes of downregulated DEMs).

To gain a deeper understanding of the molecular mechanisms by which miR-93-3p promotes wound healing through the regulation of macrophage polarization, a systematic target gene prediction analysis was carried out for the DEMs. A total of 388 target genes were predicted for the upregulated miRNAs, while 287 target genes were identified for the downregulated miRNAs (Figure 9). These high-confidence target genes offer important clues for subsequent investigations.

Through a systematic cross-analysis of DEMs among the Wugu Qilin Ointment group, Kangfuxin Solution group, and Vaseline group, our study identified five miRNAs exhibiting the most significant differential expression, along with their corresponding target genes. Specifically, as a downstream effector of mTORC1, 4E-BP1 exerts its function by modulating the translational efficiency of pro-inflammatory and anti-inflammatory cytokines through its phosphorylation status (Byles et al., 2013). miR-6889-5p inhibits EIF4EBP2, which limits macrophage protein synthesis and proliferation (Sprenkle et al., 2023). miR-378d targets RAB10 and activates the NF-κB signaling pathway to facilitate pro-inflammation mediator secretion (Zhu et al., 2020). miR-451a modulates MIF, participating in the suppression of macrophage activity and immune regulation (Li et al., 2022). miR-32-3p targets TRAF6, facilitating the ubiquitin-mediated activation of the NF-κB pathway, which drives inflammatory responses and vascular injury (Lv et al., 2018). These target genes have been corroborated by multiple studies to play direct roles in regulating MP, activating key inflammatory signaling pathways, and remodeling the wound-healing microenvironment. The present analysis not only provides theoretical support for our experimental findings from the perspective of molecular interaction networks but also further validates the biological credibility and mechanistic relevance of our screening results through established literature.

### 3.4 A DLR assay confirmed that miR-93-3p directly targets and regulates EIF4EBP1

**FIGURE 10:**
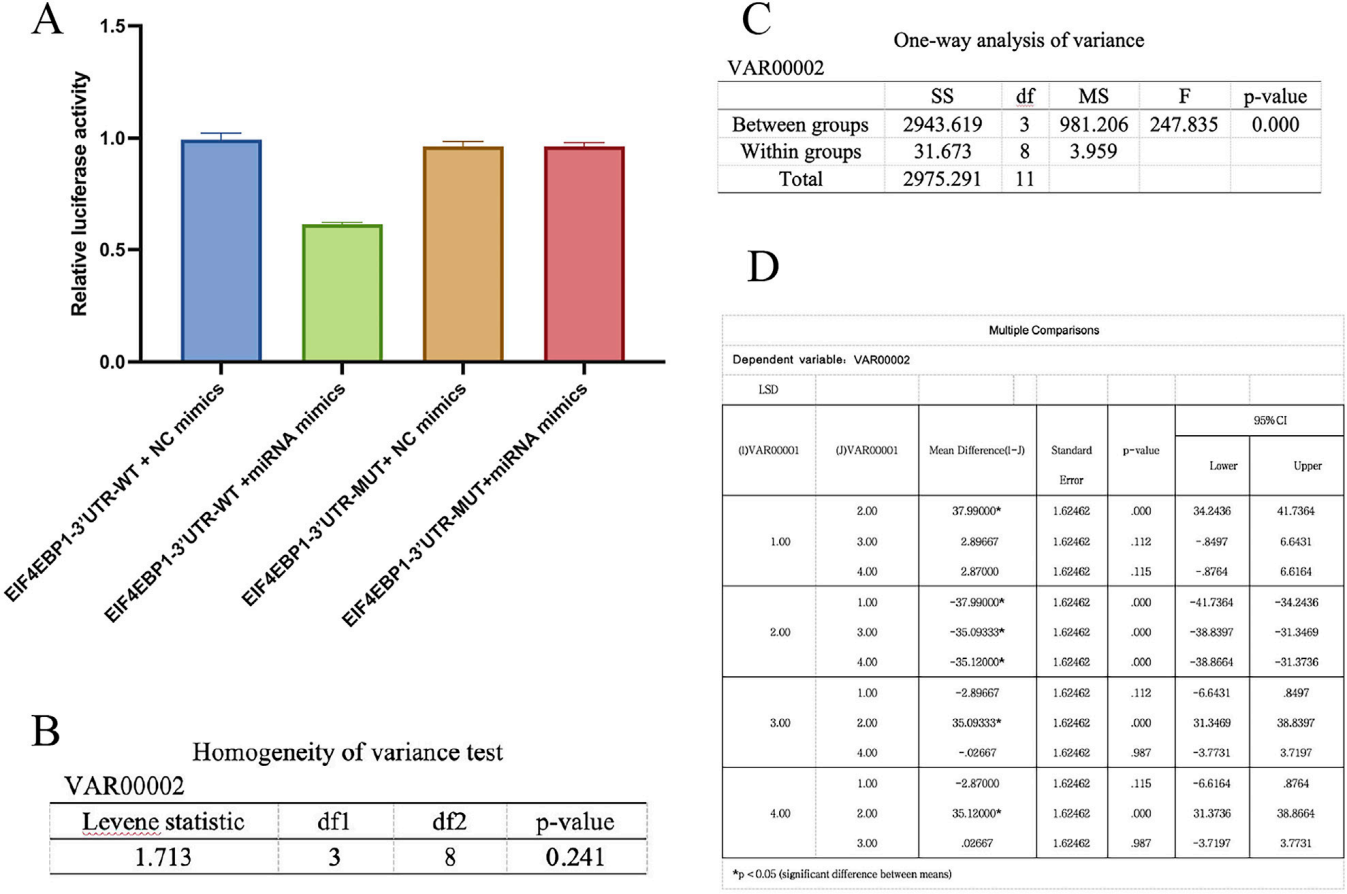
DLR Assay Validation of miR-93-3p Targeting EIF4EBP1. **(A)** Bar graph presenting DLR assay results; **(B)** Levene’s test results for homogeneity of variance; **(C)** One-way ANOVA results; **(D)** LSD post hoc comparison results.

To unveil the specific binding and mediating interaction between miR-93-3p and 3′UTR of the EIF4EBP1 gene, a rigorous functional validation was executed through a DLR system. Levene’s test for homogeneity of variance confirmed the assumption of equal variances across groups, thereby justifying subsequent analysis of variance (ANOVA). One-way ANOVA revealed a highly significant difference in luciferase activity ratios among the treatment groups. Further *post hoc* analysis using the least significant difference (LSD) method indicated that, under the wild-type 3′UTR context, luciferase activity in the miR-93-3p mimic cohort (Group B) was prominently lower than in the negative control cohort (Group A), with an inhibition rate of 63.5%. Conversely, under the mutant 3′UTR condition, the difference was insignificant across the miR-93-3p mimic cohort (Group D) and the control (Group C) (Figure 10). These results not only affirm the reliability of the experimental findings from a statistical standpoint but also substantiate, at a functional level, that miR-93-3p exerts its post-transcriptional regulatory effect by specifically binding to a target site within 3′UTR of EIF4EBP1. Mutation of this binding site completely abolished the regulatory effect. This provides direct molecular evidence supporting the therapeutic mechanism of the TCM-based “Euriching Pus for Tissue Growth” method in wound healing. The data further highlight the significance of the miR-93-3p/EIF4EBP1 axis in modulating MP and inflammatory responses, thereby offering a potential molecular target and theoretical foundation for the modernization of TCM.

### 3.5 GO and KEGG enrichment analyses of DEMs revealed potential mechanisms by which these miRNAs promote wound healing through the regulation of macrophage polarization

To unveil the possible biological functions of DEMs, systematic enrichment analyses were carried out via GO and KEGG.

**FIGURE 11:**
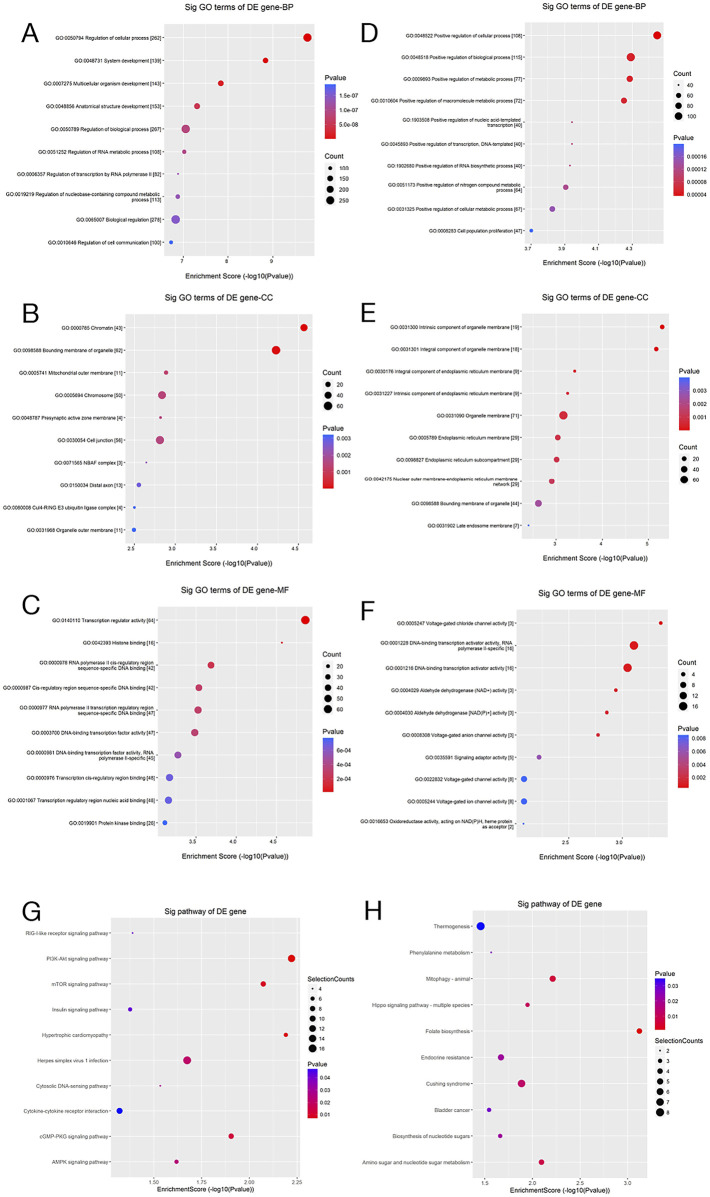
GO and Pathway Enrichment Analyses. **(A–F)** Scatter plots of GO enrichment analysis (BP, CC, and MF) for the predicted target genes of differentially upregulated and downregulated miRNAs; **(G,H)** Corresponding pathway enrichment scatter plots. The x-axis denotes the enrichment score (−log10 p-value), whereas the y-axis reflects the enriched GO terms or pathways. Dot size represents the number of enriched genes, and color depth reflects statistical significance (p-value), with red representing higher significance.

GO analysis examined gene function across three dimensions: biological process (BP), cellular component (CC), and molecular function (MF) (Figures 11A–F). Regarding BP, differentially expressed genes were notably enriched in pathways related to the regulation of inflammatory responses and MP. The CC analysis revealed that these genes are predominantly localized to the cell membrane and exosomes, while the MF analysis indicated enrichment in cytokine binding and protein kinase activity. This finding is highly consistent with the results presented in the earlier part of this study: miR-93-3p derived from wound exudate exosomes significantly upregulates the expression of M2 macrophage markers (Arg-1 and CD206) and anti-inflammatory cytokines (IL-10 and TGF-β) (P < 0.001), while concurrently suppressing the production of pro-inflammatory factors related to the M1 phenotype. This regulatory mechanism effectively promotes the phenotypic transition of macrophages from the pro-inflammatory M1 type to the anti-inflammatory M2 type and is critical in inhibiting excessive inflammatory responses.

Subsequent KEGG pathway analysis revealed marked enrichment of the target genes of DEMs in the Toll-like receptor, NF-κB, and wound healing pathways (Figures 11G,H). These findings, from a systems biology perspective, suggest that DEMs possibly contribute to wound repair by regulating key biological processes like inflammation and immune modulation. Furthermore, these signaling pathways are closely related to MP, thereby lending additional support to our hypothesis that differentially expressed exosomal miRNAs possibly inhibit inflammation and ameliorate wound healing through MP regulation.

## 4 Discussion

Through miRNA microarray analysis, our study noted significant differential expression of multiple miRNAs in the Wugu Qilin Ointment group, developed based on the “Euriching Pus for Tissue Growth” theory, in contrast to the control (|FC| > 2.0, p < 0.05). By intersecting DEMs across three groups, miR-93-3p was identified as the most significantly dysregulated candidate. Bioinformatic prediction, coupled with DLR assay validation, confirmed that miR-93-3p targets 3′UTR of EIF4EBP1 and participates in MP and inflammation regulation. This is the first study elucidating the regulatory effects of miRNA on postoperative wound healing following AF surgery.

Previous research has proved that miRNAs mediate gene expression through binding to target genes and are vital in inflammatory responses (Huntzinger and Izaurralde, 2011). For example, NKAP, as a target of miR-709, reduces inflammation through activating the NF-κB signaling pathway (Liu et al., 2020; Xiong et al., 2022). Similarly, miR-192 and its target gene BIG1 regulate MP via the PI3K/AKT/NF-κB axis (Wu et al., 2024). Our findings demonstrate that miR-93-3p directly targets 3′UTR of EIF4EBP1, consistent with prior studies showing that miR-93-3p alleviates renal injury by targeting NFAT5 (Sun et al., 2025), promotes cellular proliferation and migration via targeting ZFP36L1 (Feng et al., 2021), and modulates myocarditis by regulating TLR4 expression (Tang et al., 2018). Notably, this study is the first to report a novel mechanism by which the miR-93-3p/EIF4EBP1 axis attenuates wound inflammation through the promotion of M2-type MP.

Collectively, our findings prove for the first time that miR-93-3p directly binds to 3′UTR of EIF4EBP1, markedly inhibits its expression, and consequently promotes MP toward the M2 phenotype, thereby alleviating wound inflammation.

EIF4EBP1 (also known as 4E-BP1) is an eIF4E-binding protein and a key regulator of translation initiation. It modulates MP by influencing mRNA translational efficiency (Rolli-Derkinderen et al., 2003). In its unphosphorylated form, EIF4EBP1 binds to eIF4E, thereby repressing translation and limiting anti-inflammatory cytokine synthesis, which favors M1 polarization. In contrast, inactivation of EIF4EBP1 (e.g., via phosphorylation) relieves translational repression and promotes M2 polarization (William et al., 2018). miR-93-3p suppresses EIF4EBP1 expression by targeting its 3′UTR, thus lifting translational inhibition and facilitating M2 polarization. These findings align with prior research indicating that sustained mTORC1 signaling pathway activation, which causes EIF4EBP1 inactivation, increased secretion of TNF-α and IL-6, and reduced production of IL-10 (Byles et al., 2013). DLR assays further confirmed that mutation of the critical miR-93-3p binding site within the EIF4EBP1 3′UTR abolished this regulatory interaction, underscoring the central effects of the miR-93-3p/EIF4EBP1 axis in inflammation modulation. These findings suggest that Wugu Qilin Ointment possibly promotes wound healing through this molecular pathway. Nonetheless, this study has limitations. First, the samples in every group were limited (n = 5). Although statistical significance was achieved, expanding the sample size possibly helped uncover more subtle regulatory differences. Additionally, the downstream molecular network of EIF4EBP1 remains to be fully elucidated. Future studies should consider multicenter collaboration to increase sample size and employ techniques like single-cell sequencing to further investigate the cell-type-specific regulatory mechanisms of the miR-93-3p/EIF4EBP1 axis.

## 5 Conclusion

This study firstly systematically unravels the pivotal regulatory mechanism of the miR-93-3p/EIF4EBP1 molecular axis in wound healing. DLR assays (Figure 11A) proved miR-93-3p′s binding to 3′UTR of the EIF4EBP1 gene, which significantly downregulated its expression (the inhibitory effect was abolished upon mutation of the binding site, P < 0.001). Functional analyses further revealed that this regulatory interaction facilitates the transition from the pro-inflammation M1 (characterized by a reduction in CD86^+^ cells and decreased IL-1β secretion) to the anti-inflammation M2 subtype (marked by an increase in CD206^+^Arg-1^+^ cells and elevated TGF-β secretion). Integrative analyses of GO and KEGG pathway enrichment revealed that EIF4EBP1 functions as a central nodal molecule, orchestrating key pathways like Toll-like receptor signaling, NF-κB signaling, as well as wound healing pathways. These findings collectively delineate a comprehensive molecular network linking inflammatory modulation to tissue repair. Notably, this study is the first to unveil, at the molecular level, the scientific basis underlying the TCM practice of “Euriching Pus for Tissue Growth”. It confirms that the therapeutic efficacy of this approach is mediated via miRNA-regulated inflammation-repair homeostasis. Furthermore, our findings offer theoretical support and identify prospective treatment targets for novel wound healing strategy formulation based on targeted modulation of the miR-93-3p/EIF4EBP1 axis, underscoring their significant medical value.

## Data Availability

The original contributions presented in the study are included in the article/Supplementary Material, further inquiries can be directed to the corresponding author.
