# Supplementary material for: Exosomal miR-93-3p targets EIF4EBP1 to regulate macrophage polarization and accelerate wound healing post-anal fistula surgery
Source: Front Pharmacol. 2025 Aug 18;16:1599633. doi: 10.3389/fphar.2025.1599633 (PMC12399553; doi:10.3389/fphar.2025.1599633)
Supplement: Supplementary file 2 [file DataSheet6.docx]

RNA Quantification of Samples

| Sample ID | OD260/280  Ratio | OD260/230  Ratio | Conc.(ng/μl) | Volume(μl) | Quantity(ng) | QC Purity Pass or Fail |
| --- | --- | --- | --- | --- | --- | --- |
| Wugu group 1 | 1.70 | 1.44 | 226.62 | 15 | 3,399.30 | Pass |
| Wugu group 2 | 1.73 | 1.66 | 231.03 | 15 | 3,465.45 | Pass |
| Wugu group 3 | 1.72 | 1.68 | 306.12 | 15 | 4,591.80 | Pass |
| Wugu group 4 | 1.71 | 1.72 | 332.42 | 15 | 4,986.30 | Pass |
| Wugu group 5 | 1.73 | 1.88 | 342.51 | 15 | 5,137.65 | Pass |
| Kangfu group 1 | 1.76 | 1.99 | 232.55 | 15 | 3,488.25 | Pass |
| Kangfu group 2 | 1.74 | 1.99 | 320.11 | 15 | 4,801.65 | Pass |
| Kangfu group 3 | 1.75 | 1.94 | 317.51 | 15 | 4,762.65 | Pass |
| Kangfu group 4 | 1.73 | 1.95 | 291.02 | 15 | 4,365.30 | Pass |
| Kangfu group 5 | 1.73 | 1.93 | 253.51 | 15 | 3,802.65 | Pass |
| Vaseline group 1 | 1.74 | 1.91 | 259.6 | 15 | 3,894 | Pass |
| Vaseline group 2 | 1.74 | 2.06 | 212.38 | 15 | 3,185.7 | Pass |
| Vaseline group 3 | 1.76 | 1.96 | 251.46 | 15 | 3,771.9 | Pass |
| Vaseline group 4 | 1.75 | 2.09 | 296.29 | 15 | 4,444.35 | Pass |
| Vaseline group 5 | 1.72 | 1.93 | 286.62 | 15 | 4,299.30 | Pass |

Wugu group: Wugu Qilin Ointment group; Kangfu group: Kangfuxin Solution group
