# Supplementary material for: Exosomal miR-93-3p targets EIF4EBP1 to regulate macrophage polarization and accelerate wound healing post-anal fistula surgery
Source: Front Pharmacol. 2025 Aug 18;16:1599633. doi: 10.3389/fphar.2025.1599633 (PMC12399553; doi:10.3389/fphar.2025.1599633)
Supplement: Supplementary file 4 [file DataSheet1.doc]

**Inclusion and Exclusion Criteria**

1 Inclusion Criteria

(1) Patients diagnosed with low simple anal fistula according to Western medicine, based on the diagnostic criteria outlined in the *Clinical Diagnosis and Treatment Guidelines for Anal Fistula (2006 Edition)*;

(2) Patients diagnosed with anal fistula of the Damp-Heat downward pouring type according to Traditional Chinese Medicine (TCM), based on the syndrome differentiation criteria of “Obstruction due to Damp-Heat Accumulation” in the *Traditional Chinese Medicine Surgery (Fifth Edition)*, a national planning textbook published by China Traditional Chinese Medicine Press in 2021;

(3) Fistula tract length between 0.5 cm and 6 cm;

(4) Patients of any gender, aged between 18 and 60 years, with disease duration ranging from 3 months to 10 years;

(5) No previous history of anal surgery and no abnormalities in anal function or morphology;

(6) Undergoing low anal fistulectomy, with postoperative wounds left open (not sutured);

(7) Willing to participate in this study and having signed the informed consent form.

2 Exclusion Criteria

(1) Patients with serious diseases of vital organs such as the heart, liver, spleen, lungs, or kidneys;

(2) Patients with other anal diseases that severely affect wound healing, such as anorectal tumors;

(3) Patients with serious metabolic or immune disorders, such as diabetes or inflammatory bowel disease;

(4) Patients with infectious diseases such as HIV/AIDS, syphilis, or tuberculosis;

(5) Female patients who are menstruating, breastfeeding, or pregnant;

(6) Patients with a history of abnormal anal function or morphology;

(7) Participation in other clinical trials of new drugs within the past three months;

(8) Patients with an allergic constitution or known allergies to any component of the study medication;

(9) Patients whose anesthesia method, surgical procedure, or medication usage does not conform to the study protocol and may affect treatment efficacy.
