# Supplementary material for: Exosomal miR-93-3p targets EIF4EBP1 to regulate macrophage polarization and accelerate wound healing post-anal fistula surgery
Source: Front Pharmacol. 2025 Aug 18;16:1599633. doi: 10.3389/fphar.2025.1599633 (PMC12399553; doi:10.3389/fphar.2025.1599633)
Supplement: Supplementary file 5 [file DataSheet7.doc]

**Experimental Procedure**

**1 Isolation and Identification of Exosomes from Wound Exudate**

- 1. Isolation of Exosomes from Wound Exudate

Wound exudate samples were collected on postoperative day 7. Exosomes were isolated using ultracentrifugation. The specific steps were as follows: centrifuge the liquid samples at 500 × g for 5 minutes to remove cells, then transfer the supernatant to a new polycarbonate tube and centrifuge at 2,000 × g for 10 minutes. Collect the supernatant and transfer it to another new polycarbonate tube, then centrifuge at 10,000 × g for 30 minutes to remove shed microvesicles (sMVs, 200–1000 nm). The resulting supernatant was filtered through a 0.22 μm membrane filter and centrifuged at 100,000 × g for 70 minutes. Finally, the exosomes were resuspended in 1× PBS.

1.2 Transmission Electron Microscopy (TEM) Identification of Exosomes

(1) Plasma-clean the copper grid for 20 seconds with the carbon side facing up.

(2) Cool the sealing film on an ice box and place the copper grid on the cooled sealing film.

(3) Sequentially add 20 µl each of wound exudate-derived exosome suspension, ultrapure water, and phosphotungstic acid (PTA) staining solution onto the sealing film; let each drop stand for 1–2 minutes to allow cooling.

(4) Invert the carbon side of the copper grid onto the exosome suspension and incubate for 1 minute for adsorption.

(5) Wick away excess liquid for 10 seconds until no visible residue remains.

(6) Invert the carbon side of the copper grid onto ultrapure water and repeat steps (4) and (5).

(7) Invert the carbon side of the copper grid onto the PTA droplet and repeat steps (4) and (5).

(8) Place the grid on filter paper, avoid direct sunlight, and allow it to air-dry in a cool, shaded place before observing under TEM.

1.3 Extraction of Total RNA from Exosomes

(1) Thaw the isolated and purified exosome samples and centrifuge at 12,000 × g for 10 minutes to remove impurities. Mix 250 µl of the sample with 750 µl of TRIzol LS Reagent.

(2) Add 0.2 ml of chloroform per 750 µl of TRIzol LS homogenized sample, cap, and shake vigorously, then incubate at 15–30°C for 4–5 minutes. Centrifuge at 12,000 × g for 15 minutes.

(3) Add 500 µl of isopropanol to the aqueous phase, mix well to precipitate RNA, then incubate at 15°C for 15 minutes and centrifuge at 12,000 × g for 10 minutes.

(4) Remove the supernatant, add 1 ml of 75% ethanol, and centrifuge at 7,500 × g for 10 minutes.

(5) Discard the ethanol, air-dry the RNA pellet for 5 minutes, then resuspend in RNase-free water by pipetting several times. Incubate at 50°C for 15 minutes. Store the RNA solution at –70°C.

**2 miRNA Microarray Detection**

(1) Add 500 ng of total RNA to a centrifuge tube and mix with 2 μL of calf intestinal phosphatase mixture. Incubate at room temperature for 20 minutes, then add 2.8 μL of DMSO and incubate at 90°C for 10 minutes. Add 4.5 μL of Ligation Master Mix and incubate at 16°C for 1 hour.

(2) Resuspend the dried sample in 18 μL of nuclease-free water. Add 4.5 μL of 10× Gene Expression Blocking Agent and 2× Hi-RPM Hybridization Buffer, incubate at 100°C for 5 minutes, and then immediately place on ice for 5 minutes. Place the assembled slide chamber into a hybridization oven at 55°C, rotating at 20 rpm, and hybridize at 55°C for 20 hours.

(3) Add 2 mL of 10% Triton S-102 to the wash buffer container and invert the container 5–6 times to mix. Preheat staining dish #3 with a sufficient amount of Gene Expression Wash Buffer to 37°C. Fill the staining dish with 100% acetonitrile, turn on the magnetic stirrer, set it to medium speed (level 4), and wash for 5 minutes. Discard the acetonitrile. At room temperature, fill slide staining dish #1 with Wash Buffer 1, and add enough Wash Buffer 1 to slide staining dish #2 to cover the slide rack. Preheat a 1.5 L glass dish filled with water and install staining dish #3 on a magnetic stirrer with a heating element. Add about three-quarters of Wash Buffer 2 to slide staining dish #3 and place a magnetic stir bar inside to maintain the temperature of Wash Buffer 2 at room temperature. Place the sandwich into Gene Expression Wash Buffer 1 and open it from the edge. After separating the slides within the group, place all slides into the side rack of staining dish #2. Then transfer the slide rack into staining dish #3 containing Wash Buffer 2 and stir at medium speed for 5 minutes. Finally, perform chip scanning and data extraction.

**Main Reagents and Instruments**

1 Main Reagents

Table 1 Main Reagents

| Reagent/Consumable | Manufacturer | Catalog No. |
| --- | --- | --- |
| 0.22 um membrane filter | Merck Millipore |  |
| TRIzol LS Reagent | Invitrogen | 10296028 |
| miRNA Complete Labeling and Hyb Kit | Agilent | 5190-0456 |
| RT primers | [Shanghai Generay](http://www.baidu.com/link?url=3SYYoHsQfdu7kMx9GBs13JbjPq1r4lxprcq4pbhBzQIg4tBziTWqknU0qy24hD01Ik7shtxUtpy6ELkFYdzNJ_) |  |
| 100% Ethanol | Shanghai Chemical |  |
| PBS | Biosharp | BL551A |
| Formaldehyde loading dye | Invitrogen |  |
| EDTA | CUSABIO |  |
| M-MuLV Reverse Transcriptase | Enzymatics | P7040L |
| Sodium acetate | Shanghai Chemical |  |
| Formaldehyde | CUSABIO |  |
| Gene Expression Wash Buffer Kit | Agilent | 5188-5327 |
| GelRed | Biotium | 41003 |
| Agarose | Sangon |  |
| Chloroform | Shanghai Chemical |  |
| Tris-HCI | CUSABIO |  |
| RNase lnhibitor | Enzymatics | Y9240L |
| MOPS | CUSABIO |  |
| dNTP Mix | HyTest Ltd |  |
| lsopropanol | Shanghai Chemical |  |
| 2X PCR Master Mix | Arraystar | AS-MR-006-5 |
| Glycogen | Invitrogen | AM9510 |

2 Main Instruments

Table 2 Main Instruments

| Instrument | Manufacturer | Model |
| --- | --- | --- |
| Hybridization Chamber, stainless | Agilent | G2534A |
| Hybridization Chamber gasket slides | Agilent | G2534-60003 |
| Hybridization oven | Agilent | G2545A |
| Hybridization Chambers | Agilent | G2530-60029 |
| Microarray Scanne | Agilent | G2505C |
| Gene Amp PCR System 9700 | Applied Biosystems |  |
| QuantStudio5 Real-time PCR System | Applied Biosystems |  |
