# Supplementary material for: Exosomal miR-93-3p targets EIF4EBP1 to regulate macrophage polarization and accelerate wound healing post-anal fistula surgery
Source: Front Pharmacol. 2025 Aug 18;16:1599633. doi: 10.3389/fphar.2025.1599633 (PMC12399553; doi:10.3389/fphar.2025.1599633)
Supplement: Supplementary file 7 [file DataSheet10.doc]

**Experimental Procedure**

**Dual-luciferase Reporter Assay**

1 Cell Thawing

293T cell culture medium: DMEM + 10% FBS + 1% Penicillin-Streptomycin Solution.

(1) Quickly remove 293T cells from liquid nitrogen and place them in a 37°C water bath; gently shake the cryovial until the freezing medium is thawed.

(2) Transfer the thawed cells to a centrifuge tube containing 5 ml culture medium, centrifuge at 1000 rpm for 5 minutes at room temperature, and discard the supernatant.

(3) Resuspend cells in a complete medium containing 10% FBS and seed into culture dishes. Gently pipette to mix. Incubate at 37°C with 5% CO₂ in a humidified incubator.

2 Cell Passage

When cell density reaches 80%, passage the cells as follows:

(1) Discard the medium and wash once with PBS.

(2) Add 1 ml trypsin and digest for 2 minutes under the microscope until cells become round and detached.

(3)Quickly discard the trypsin, add complete medium, gently pipette to make a single-cell suspension, and passage at a ratio of 1:3. Incubate at 37°C, 5% CO₂ in a humidified incubator.

3 Cell Transfection

(1) Seed healthy 293T cells in the logarithmic growth phase at a density of 1×10⁵ cells per well in a 12-well plate, and culture overnight in a 37°C, 5% CO₂ incubator.

(2) Two hours before transfection, replace the medium with serum-free DMEM.

(3) Transfection procedure:

For each transfection sample, prepare as follows:

a) Dilute 1 μg plasmid and 2.5 μl siRNA in 50 μl serum-free opti-MEM, mix gently, and incubate at room temperature for 5 minutes.

b) Gently mix Lipofectamine™ 2000 before use, then dilute 3 μl Lipofectamine™ 2000 in 50 μl opti-MEM and incubate at room temperature for 5 minutes.

c) Mix the diluted Lipofectamine™ 2000 with the plasmid and siRNA mixture (final volume 100 μl), mix gently, and incubate at room temperature for 20 minutes.

(4) Add 100 μl of the mixture to each well and gently shake the plate to mix evenly.

(5) Incubate cells at 37°C in a CO₂ incubator. After 6 hours, replace the transfection mixture with a normal culture medium.

(6) Continue incubation at 37°C in a 5% CO₂ humidified incubator.

4 Cell Treatment

(1) Logarithmically growing, healthy 293T cells were seeded into 12-well plates at a density of 1 × 10⁵ cells per well and incubated overnight at 37°C in a 5% CO₂ incubator.

(2) According to the transfection procedure described above, the cells were treated as follows:

Group A: EIF4EBP1-3’UTR wild-type + NC mimics

Group B: EIF4EBP1-3’UTR wild-type + miRNA mimics

Group C: EIF4EBP1-3’UTR mutant-type + NC mimics

Group D: EIF4EBP1-3’UTR mutant-type + miRNA mimics

Incubation time: Cells were cultured for an additional 48 hours post-transfection.

5 Dual-luciferase Reporter Assay

(1) After the required culture period, the culture medium was aspirated, and the cells were rinsed once with PBS. Then, 300 μl of reporter lysis buffer was added

(2) After complete lysis, the lysate was centrifuged at 12,000 rpm for 3 minutes. The supernatant was collected for analysis.

(3) Firefly luciferase assay reagent and Renilla luciferase assay buffer were thawed and brought to room temperature. The Renilla luciferase substrate (100X) was kept on ice for use.

(4) Prepare Renilla luciferase working solution by mixing Renilla assay buffer with the Renilla substrate (100X) at a 1:100 ratio, according to a volume of 100 μl per sample.

(5) Add 100 μl of the sample lysate to 100 μl of firefly luciferase reagent, mix thoroughly, and measure the relative light units (RLU).

(6) Then add 100 μl of Renilla luciferase working solution, mix thoroughly, and measure the RLU again.

Using firefly luciferase as the internal control, divide the RLU value obtained from the Renilla luciferase assay by the RLU value from the firefly luciferase assay. This ratio reflects the relative activation level of the target reporter gene across different samples.**Main Reagents and Instruments**

1Main Instruments

Table 1 Main Instruments

| Name | Manufacturer | Model |
| --- | --- | --- |
| Micropipette | sartorius/dragonlab |  |
| Ultra-clean Workbench | LAYTE | SW-CJ-1FDG |
| COz Incubator | Shanghai Yiheng | BPN-150CH(UV) |
| Inverted Microscope | Nikon | Ta2-FL |
| Benchtop Low-speed Centrifuge | Kecheng | L3-5K |
| Benchtop High-speed Centrifuge | Kecheng | H1-16K |
| Constant Temperature Water Bath | Changzhou Aohua Instrument | HH-2 |
| Multi-mode Plate Reader | Perkins Elmer | Envision |

2 Main Reagents

Table 2 Main Reagents

| Name | Manufacturer | Model |
| --- | --- | --- |
| DMEM High Glucose Basal Medium | Keycell | QS-S011 |
| Fetal Bovine Serum | INTL KANG | F800821 |
| Penicillin-Streptomycin Solution (100x) | Keycell | QS-S402 |
| Trypsin-EDTA Solution (0.25% trypsin, with EDTAphenol red-free) | Keycell | QS-S401 |
| Phosphate-Buffered Saline (1x PBS) | Keycell | QS-S001 |
| Opti-MEM®I(1X)+ GlutaMAX™m_1 | GIBCO | 51985-034 |
| Lipofectamine 2000 | Invitrogen | 11668-019 |
| Dual-Luciferase Reporter Assay Kit | Beyotime | RG027 |
| Cell Culture Dishes and Flasks | NEST |  |
| Other Chemical Reagents | Domestically Produced Analytical Grade Reagents |  |
