# Supplementary figures and images for: Exosomal miR-93-3p targets EIF4EBP1 to regulate macrophage polarization and accelerate wound healing post-anal fistula surgery
Source: Front Pharmacol. 2025 Aug 18;16:1599633. doi: 10.3389/fphar.2025.1599633 (PMC12399553; doi:10.3389/fphar.2025.1599633)

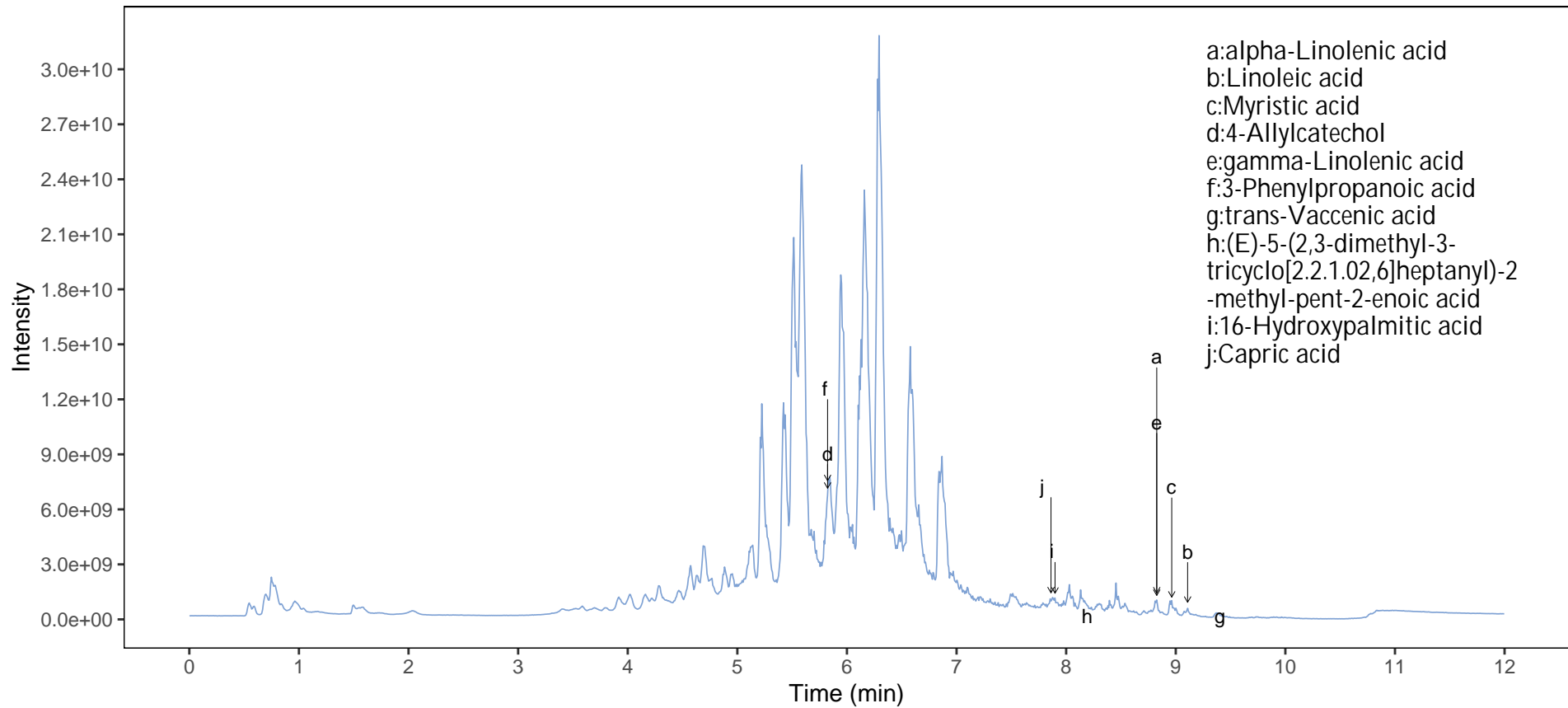

Supplement: Supplementary file 8 [file Image2.pdf]

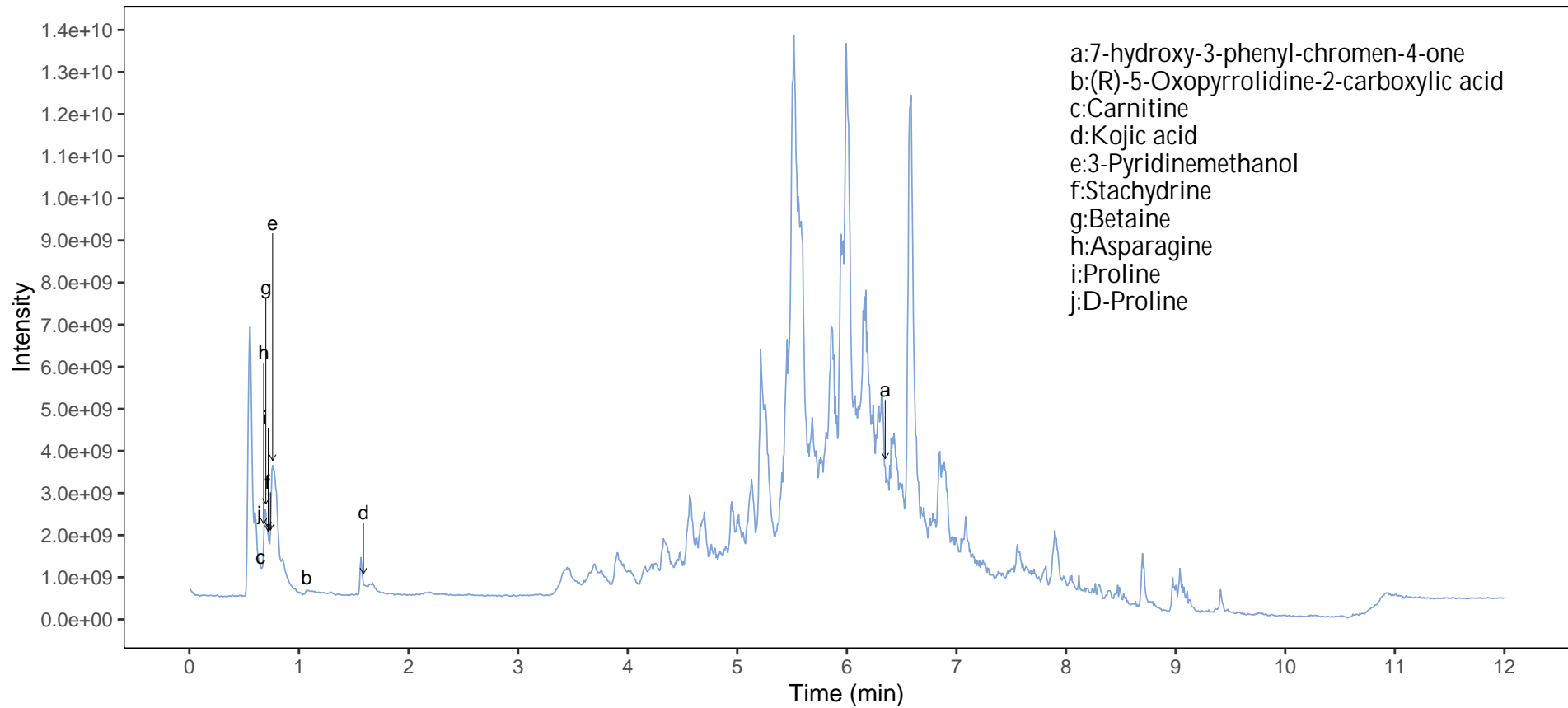

Supplement: Supplementary file 14 [file Image1.pdf]
