# Supplementary material for: Exosomal miR-93-3p targets EIF4EBP1 to regulate macrophage polarization and accelerate wound healing post-anal fistula surgery
Source: Front Pharmacol. 2025 Aug 18;16:1599633. doi: 10.3389/fphar.2025.1599633 (PMC12399553; doi:10.3389/fphar.2025.1599633)
Supplement: Supplementary file 11 [file DataSheet9.doc]

**Experimental Procedure**

**1 Cell Culture and Induction**

1.1 Cell Thawing

Retrieve the human peripheral blood mononuclear cell line THP-1 (purchased from Biotao Biotech) from liquid nitrogen. Centrifuge to collect the cells (1000 rpm, 10 minutes at room temperature). Resuspend the cells in a complete medium containing 10% fetal bovine serum and incubate at 37°C with 5% CO₂ under saturated humidity conditions.

1.2 Cell Passage

When cell density reaches 80%, passage the cells at a ratio of 1:3.

1.3 Cell Transfection

Select THP-1 cells in good growth condition. Dilute 10 μL siRNA in 100 μL serum-free Opti-MEM. Dilute 5 μL Lipofectamine™ 2000 in 100 μL Opti-MEM. Mix the diluted Lipofectamine™ 2000 with the diluted siRNA and let stand at room temperature for 15 minutes. Add 200 μL of the mixture to each well. Incubate the cells at 37°C in a CO₂ incubator. After 4 hours, remove the mixture and replace it with a normal culture medium.

**2 Real-time qPCR Detection of miR-93-3p Expression in Macrophages**

1. Use the Trizol method to extract RNA from the samples. Reverse transcribe the total RNA from exosomes into cDNA.

Table Reverse Transcription Reaction System

Table 1-1 RT1: Genomic DNA Removal (42°C for 2 min)

| Reagent | Volume (μL) |
| --- | --- |
| Template RNA | 1.758 μg |
| 4 × gDNA wiper Mix | 4 |
| Oligo (dT)23VN /loop primer (10 μM) | 1 |
| RNase-free ddH2O | Up to 16 |

Table 1-2 RT2: Reverse Transcription Reaction System

| Reagent | Volume (μL) |
| --- | --- |
| 5 × HiScript II Select qRT SuperMix II | 4 |
| RT1 Reaction Product | 16 |

Table 1-3 Reaction Conditions

| Temperature |  |
| --- | --- |
| 50℃ | 15 min |
| 85℃ | 5 s |
| 4℃ | 10 min |

(2) Real-time qPCR detection: Follow the qPCR protocol and reaction system. Use U6 as the internal control. Each reaction is performed in triplicate. The PCR reaction is run on a real-time qPCR instrument. Fluorescence signals are collected and melting curve analysis is performed.

Table 2 Real-time qPCR Reaction System

| cDNA | 4μl |
| --- | --- |
| Forward Primer (10μM) | 0.4μl |
| Reverse Primer (10μM) | 0.4μl |
| 2*Q3 SYBR qPCR Master Mix | 10μl |
| Nuclease-free Water | 5.2μl |

Conduct the PCR reaction on a real-time PCR instrument using the following cycling conditions: 95°C for 10 minutes; followed by 40 PCR cycles of 95°C for 10 seconds and 60°C for 60 seconds.

Table 3 Primer Sequences

| Name | Primer | Sequence | |
| --- | --- | --- | --- |
| U6 | Forward | | CGCTTCGGCAGCACATATAC |
|  | Reverse | AAATATGGAACGCTTCACGA | |
| Hsa-miR-93-3p | Loop Primer | GTCGTATCCAGTGCAGGGTCCGAGGTA  TTCGCACTGGATACGACCGGGAAGT | |
|  | Forward | TGCGCACTGCTGAGCTAGCAC | |

**3 Flow Cytometry Analysis of Surface Markers on Macrophages from Different Treatment Groups**

Collect the cells, and for each flow cytometry tube, prepare 1 mL PBS containing 0.5% bovine serum albumin (BSA) and 1×10⁶ cells. Centrifuge at 1500 rpm for 5 minutes and discard the supernatant. Add 1 mL of Fix/Perm working solution, mix gently, and incubate in the dark at 4°C for 50 minutes. Add 2 mL of Perm/Wash working solution to each tube, centrifuge at 1500 rpm for 3 minutes, and discard the supernatant. Resuspend the cells in 100 μL Perm/Wash working solution and add the corresponding antibody (Arginase I or CD206). Incubate in the dark at 4°C for 40 minutes. Add 2 mL of Perm/Wash working solution to each tube, centrifuge at 1500 rpm for 3 minutes, and discard the supernatant. Resuspend the cells in 0.2 mL PBS containing 0.5% BSA and proceed to flow cytometry analysis.

**4 qPCR Detection of Macrophage Markers and Inflammatory Cytokines in Different Treatment Groups**

Harvest macrophages from each treatment group. Extract total RNA using the Trizol method and perform quality control. Reverse transcribe the extracted RNA into cDNA, set up the reaction system, and follow the protocol as described in the manufacturer’s instructions. Use β-actin as the internal control. Determine the relative expression levels of M1 macrophage markers (CD86) and pro-inflammatory cytokines (IL-1β, IL-6, TNF-α), as well as M2 macrophage markers (Arg-1, CD206) and anti-inflammatory cytokines (IL-10, TGF-β).

Table 4 Primer Sequences

| Name | Primer | Sequence |
| --- | --- | --- |
| Homo β-actin | Forward | CCCTGGAGAAGAGCTACGAG |
|  | Reverse | CGTACAGGTCTTTGCGGATG |
| Homo CD86 | Forward | CCAGACCACATTCCTTGGAT |
|  | Reverse | TTAAAAACACGCTGGGCTTC |
| Homo IL-1β | Forward | CGAATCTCCGACCACCACTA |
|  | Reverse | AGCCTCGTTATCCCATGTGT |
| Homo IL-6 | Forward | AGGAGACTTGCCTGGTGAAA |
|  | Reverse | CAGGGGTGGTTATTGCATCT |
| Homo TNF-α | Forward | TCAGAGGGCCTGTACCTCAT |
|  | Reverse | GGAAGACCCCTCCCAGATAG |
| Homo Arg-1 | Forward | CCCTTTGCTGACATCCCTAA |
|  | Reverse | GACTCCAAGATCAGGGTGGA |
| Homo CD206 | Forward | CAAAAAGGGCAACACCACTT |
|  | Reverse | AGTCCAATTCCTCGATGGTG |
| Homo IL-10 | Forward | GTCCTCCTGACTGGGGTGAGG |
|  | Reverse | TTGATGTCTGGGTCTTGGTTC |
| Homo TGF-β | Forward | CAGCAACAATTCCTGGCGATACCT |
|  | Reverse | CGCTAAGGCGAAAGCCCTCAAT |

**5 Detection of Macrophage Marker Protein Expression in Different Treatment Groups by Western Blot**

Collect cells and detect the expression of M1 macrophage marker protein CD86 and M2 macrophage marker proteins Arg-1 and CD206 using the Western blot method.

5.1 Preparation of Protein Samples

(1) Protein Extraction: Lyse the cells on ice for 15 minutes using RIPA lysis buffer, centrifuge at 12,000 rpm for 10 minutes at 4°C, and collect the supernatant.

(2) Protein Quantification: Determine the protein concentration using a BCA protein assay kit.

(3) Protein Denaturation: Mix the extracted protein suspension with 5× loading buffer and denature in boiling water.

5.2 Electrophoresis: Perform electrophoresis at a constant voltage of 80V until the bromophenol blue indicator reaches the boundary between the stacking gel and separating gel, then switch to a constant voltage of 120V.

5.3 Membrane Transfer: Transfer the gel onto an activated PVDF membrane. The transfer conditions are: CD86, Arg-1, and β-actin—420 seconds at 1.5 A; CD206—600 seconds at 1.5 A.

5.4 Immunoblot Detection

(1) Blocking: Incubate the PVDF membrane in a blocking buffer on a shaker at room temperature for 1.5 hours.

(2) Primary Antibody Incubation: Dilute the corresponding primary antibodies with blocking buffer and incubate the PVDF membrane overnight at 4°C.

(3) Secondary Antibody Incubation: Dilute the HRP-conjugated secondary antibody, and incubate the membrane at room temperature on a shaker for 1.5 hours.

(4) Detection: Mix the ECL reagent, apply it to the membrane, and detect signal and image using a chemiluminescence imaging system.

**Main Reagents and Instruments**

1 Main Reagents

Table 5 Main Reagents

| Reagent/Consumable | Manufacturer | Catalog No. |
| --- | --- | --- |
| Trizol | Thermo Fisher Scientific | 15596-026 |
| PBS | Biosharp | BL551A |
| 2*Q3 SYBR qPCR Master Mix | TOLOBIO | 22204 |
| Absolute Ethanol | Sinopharm | 10009218 |
| THP-1 Cell Culture Medium | Pricella | CM-0233 |
| Phosphate Buffered Saline (1X PBS) | Keycell | QS-S001 |
| Tris-Base | Saiguo Biotech | 1115GR500 |
| PE anti-human Arginase l Antibody | BioLegend | 369703 |
| FITC anti-human CD206 (MMR) Antibody | BioLegend | 321103 |
| Phosphatase Inhibitor | servicebio | G2007 |
| PMSF | servicebio | G2008 |
| RIPA Lysis Buffer | servicebio | G2002 |
| BCA Protein Assay Kit | GBCBIO | G3522 |
| BSA Standard | GBCBIO | G8532 |
| TEMED | servicebio | GC203001 |
| 5x Protein Loading Buffer30% Acrylamide | servicebio | G2075 |
| TBST | servicebio | G2004 |
| HiScript® ll Q Select RT SuperMix for qPCR | servicebio | G0004 |
| Tween-20 | VAZYME | R233 |
| Glycerol | Sinopharm | 30189328 |
| Acetic Acid | Sinopharm | 10010618 |
| BSA Standard | Sinopharm | 10000218 |
| 4×Tris-HCL-SDS, PH8.8 | Sangon Biotech | C526033-0001 |
| Mops | Macklin | M813154 |
| PMA | sigma | P1585 |
| Glycine | Saiguo Biotech | 1275GR500 |
| SDS | Sinopharm | 30166428 |
| Protein Marker (8-180 kDa) | Yeasen | 20350ES90 |
| Protein Marker (10-120 kDa) | GenScript | M00521 |
| PVDF Membrane (0.45 μm) | Millipore | IPVH00010 |
| Mouse Monoclonal Anti-B-actin | Affinity | T0022 |
| Rabbit Polyclonal Anti-Arg1 (36 kDa) | Proteintech | 16001-1-AP |
| Mouse Monoclonal Anti-CD206 (170 kDa) | Proteintech | 60143-1-Ig |
| Rabbit Polyclonal Anti-CD86 (72 kDa) | Proteintech | 13395-1-AP |
| HRP-Conjugated Goat Anti-Rabbit lgG | Beyotime | A0208 |
| HRP-Conjugated Goat Anti-Mouse lgG | Proteintech | SA00001-1 |
| ECL Substrate Solution | servicebio | G2014 |

2 Main Instruments

Table 6 Main Instruments

| Instrument | Manufacturer | Model |
| --- | --- | --- |
| Tissue Grinder | Shanghai Jingxin | Tissuelyser-24L |
| Benchtop High-speed Refrigerated Centrifuge | Hunan Kecheng Instrument and Equipment | HI-16KR |
| Benchtop High-speed Centrifuge | Hunan Kecheng Instrument and Equipment | HI-16K |
| Magnetic Stirrer with Heating | Shanghai Meixiang Instrument | CJJ78-1 |
| Gel lmaging System | Beijing Jiuyi Instrument | WD-9413B |
| Electrophoresis Apparatus | Beijing Jiuyi Instrument | OYY-6C |
| Vortex Mixer | Haimen Kylin-Bell Lab Instruments | QL-901 |
| Ultranano UV-Visible Spectrophotometer | MIULAB | ND-100 |
| Mini Centrifuge | lahyeah |  |
| Real-Time PCR System | Thermo Fisher Scientific | ViiA-7 |
| qPCR Tubes | EXTRAGENE / illumina |  |
| PCR Amplifier | ABI | proFlex |
| Aquapro Ultrapure Water System | Aquapro | AJY -0501 |
| CO_2_ Incubator | Shanghai Yiheng | BPN-150CH(UV) |
| Inverted Microscope | Nikon | Ta2-FL |
| Benchtop Low-Speed Centrifuge | Hunan Kecheng Instrument and Equipment | L3-5K |
| Constant Temperature Water Bath | Changzhou Aohua Instrument | HH-2 |
| Flow Cytometer | BECKMAN | CytoFLEX |
| Vertical Electrophoresis Cel | Beijing Liuyi Instrument Factory | DYCZ-24DN |
| Vertical Electrophoresis Cell | WIX | WIX-easyPRO4 |
| Electrophoretic Transfer Unit | Beijing Liuyi Instrument Factory | DYCZ-40 |
| Semi-dry Transfer Apparatus | Nanjing Aisiyi | FW606 |
| Microplate Reader | BIO-Tek | MQX200 |
| Analytical Balance | Radwag | AS220.X2 |
