# Supplementary material for: Exosomal miR-93-3p targets EIF4EBP1 to regulate macrophage polarization and accelerate wound healing post-anal fistula surgery
Source: Front Pharmacol. 2025 Aug 18;16:1599633. doi: 10.3389/fphar.2025.1599633 (PMC12399553; doi:10.3389/fphar.2025.1599633)
Supplement: Supplementary file 12 [file DataSheet8.docx]

qPCR verification of differentially expressed miRNAs

1. Total RNA from samples is extracted using the Trizol method, and the isolated exosomal RNA is reverse-transcribed into cDNA. The specific steps are as follows:
   1. cDNA Synthesis

Table 1-1 RT Reaction Mixture Preparation System

| Reagent | Volume |
| --- | --- |
| dNTP | 2ul |
| 10X RT Buffer | 2ul |
| RT-specific primer | 0.3ul |
| Total RNA | 800ng |
| M-MuLV Reverse Transcriptase | 0.2ul |
| RNase inhibitor | 0.3ul |
| Nuclease-free water | Up to 20ul |

- 1. Perform the RT reaction using a PCR thermal cycler under the following conditions: 16° C for 30 minutes; 42°C for 40 minutes; 85°C for 15 minutes. After the reaction is complete, place the reaction product on the ice for immediate use or store it at -20°C.

1. Follow the qPCR procedure and reaction system for the operation. Use the U6 gene as an internal control, and set up three technical replicates for each reaction. Perform PCR reactions on a real-time PCR instrument. Fluorescence signals are collected and melting curve analysis is conducted. The relative expression levels of differentially expressed miRNAs are calculated using the 2^-ΔΔCT method. The specific steps are as follows:
2. Real-time qPCR primer information:

Table 1-2 miRNA Primers Used for Real-time qPCR Detection

| Gene Name | Forward and Reverse Primer Sequences |
| --- | --- |
| U6 | F:5’GCTTCGGCAGCACATATACTAAAAT3’  R:5’CGCTTCACGAATTTGCGTGTCAT3’ |
| hsa-miR-32-3p | GSP:5’GGGGGCAATTTAGTGTGTGT3’  R:5’GTGCGTGTCGTGGAGTCG3’ |
| hsa-miR-3663-3p | GSP:5’GGATGAGCACCACACAGGC3’  R:5’GTGCGTGTCGTGGAGTCG3’ |
| hsa-miR-378d | GSP:5’GGGGAACTGGACTTGGAG3’  R:5’GTGCGTGTCGTGGAGTCG3’ |
| hsa-miR-4635 | GSP:5’GGGGGTCTTGAAGTCAGAAC3’  R:5’GTGCGTGTCGTGGAGTCG3’ |
| hsa-miR-6507-3p | GSP:5’GGGGCAAAGTCCTTCCTATT3’  R:5’GTGCGTGTCGTGGAGTCG3’ |
| hsa-miR-6769a-5p | GSP:5’GGAAAGGTGGGTATGGAGG3’  R:5’GTGCGTGTCGTGGAGTCG3’ |
| hsa-miR-6889-5p | GSP:5’TCGGGGAGTCTGGGGTC3’  R:5’GTGCGTGTCGTGGAGTCG3’ |
| hsa-miR-762 | GSP:5’GGGGCTGGGGCCGG3’  R:5’GTGCGTGTCGTGGAGTCG3’ |

| hsa-miR-135b-3p | GSP:5’GGGGGATGTAGGGCTAAAAG3’  R:5’GTGCGTGTCGTGGAGTCG3’ |
| --- | --- |
| hsa-miR-93-3p | GSP:5’GGGACTGCTGAGCTAGCAC3’  R:5’GTGCGTGTCGTGGAGTCG3’ |
| hsa-miR-378g | GSP:5’GGGAAACTGGGCTTGGAG3’  R:5’GTGCGTGTCGTGGAGTCG3’ |
| hsa-miR-15a-5p | GSP:5’GGGTAGCAGCACATAATGG3’  R:5’CAGTGCGTGTCGTGGAGT3’ |
| hsa-miR-451a | GSP:5’GGGGGAAACCGTTACCATTAC3’  R:5’GTGCGTGTCGTGGAGTCG3’ |

1. Real-time qPCR procedure:

Prepare the real-time PCR reaction system for each cDNA sample as follows: Table 1-3 Realtime PCR Reaction System

| Reagent | Volume |
| --- | --- |
| 2 × Master Mix | 5 µl |
| PCR-specific Primer F | 0.5µl |
| PCR-specific Primer R | 0.5µl |
| Nuclease-free Water | Up to 8ul |

Conduct the PCR reaction on a real-time PCR instrument using the following cycling conditions: 95°C for 10 minutes; followed by 40 cycles of 95°C for 10 seconds and 60°C for 60 seconds.
