# Supplementary material for: Exosomal miR-93-3p targets EIF4EBP1 to regulate macrophage polarization and accelerate wound healing post-anal fistula surgery
Source: Front Pharmacol. 2025 Aug 18;16:1599633. doi: 10.3389/fphar.2025.1599633 (PMC12399553; doi:10.3389/fphar.2025.1599633)
Supplement: Supplementary file 13 [file DataSheet2.doc]

Table 1. Major Medicinal Ingredients of Wugu Qilin Ointment and Their Taxonomic Verification

| Latin Name | Family and Genus | Medicinal Part | Species Identification by Taxonomic Database |
| --- | --- | --- | --- |
| Chrysomyia megacephala (Fabricius) | Calliphoridae, Chrysomyia genus | Dry larva | The “Zhejiang Province Traditional Chinese Medicine Processing Standards” (2022 Edition): “Wuguchong”. |
| Daemonorops draco Bl | Arecaceae | The resin exuded from the fruit | Chinese Pharmacopoeia (2020 Edition): “XueJie”. |
| Coptis chinensis Franch | Ranunculaceae | Dried rhizomes | Chinese Pharmacopoeia (2020 Edition) : “WeiLian”. |
| Arnebia euchroma (Royle) Johnst | Boraginaceae | Dried root | Chinese Pharmacopoeia (2020 Edition): “ZiCao”. |

Orthogonal fingerprint analysis was performed via liquid chromatography-mass spectrometry (LC-MS) in both positive and negative ion modes. Based on the scoring values, the top 10 characteristic peaks were selected for total ion chromatogram (TIC) analysis. Tables 2 and 3 present the detailed data:

Table 2. TIC Reference Peaks in Positive Ion Mode

| ID | MS2_name | Formula | mzmed | rtmed |
| --- | --- | --- | --- | --- |
| 1 | 7-hydroxy-3-phenyl-chromen-4-one | C15H10O3 | 239.0703 | 381 |
| 2 | (R)-5-Oxopyrrolidine-2-carboxylic acid | C5H7NO3 | 130.0499 | 64.2 |
| 3 | Carnitine | C7H15NO3 | 162.1125 | 39.3 |
| 4 | Kojic acid | C6H6O4 | 143.0339 | 95.3 |
| 5 | 3-Pyridinemethanol | C6H7NO | 110.06 | 45.6 |
| 6 | Stachydrine | C7H13NO2 | 144.1019 | 44.4 |
| 7 | Betaine | C5H11NO2 | 118.0862 | 41.9 |
| 8 | Asparagine | C4H8N2O3 | 133.0608 | 40.7 |
| 9 | Proline | C5H9NO2 | 116.0707 | 43.2 |
| 10 | D-Proline | C5H9NO2 | 116.0707 | 43.2 |

Table 3. TIC Reference Peaks in Negative Ion Mode

| ID | MS2_name | Formula | mzmed | rtmed |
| --- | --- | --- | --- | --- |
| 1 | alpha-Linolenic acid | C18H30O2 | 277.2173 | 529.6 |
| 2 | Linoleic acid | C18H32O2 | 279.233 | 546.5 |
| 3 | Myristic acid | C14H28O2 | 227.2016 | 537.8 |
| 4 | 4-Allylcatechol | C9H10O2 | 149.0608 | 349.4 |
| 5 | gamma-Linolenic acid | C18H30O2 | 277.2173 | 529.6 |
| 6 | 3-Phenylpropanoic acid | C9H10O2 | 149.0608 | 349.4 |
| 7 | trans-Vaccenic acid | C18H34O2 | 281.2487 | 564.1 |
| 8 | (E)-5-(2,3-dimethyl-3-tricyclo[2.2.1.02,6]heptanyl)-2-methyl-pent-2-enoic acid | C15H22O2 | 233.1549 | 491.5 |
| 9 | 16-Hydroxypalmitic acid | C16H32O3 | 271.228 | 473.9 |
| 10 | Capric acid | C10H20O2 | 171.1391 | 471.7 |
